# Identification of 10 genes on *Candida albicans* chromosome 5 that control surface exposure of the immunogenic cell wall epitope β-glucan and cell wall remodeling in caspofungin-adapted mutants

**DOI:** 10.1128/spectrum.03295-23

**Published:** 2023-11-15

**Authors:** Sudisht K. Sah, Anshuman Yadav, Michael D. Kruppa, Elena Rustchenko

**Affiliations:** 1 Department of Biochemistry and Biophysics, University of Rochester Medical Center, Rochester, New York, USA; 2 Department of Biomedical Sciences, Center of Excellence in Inflammation, Infectious Disease and Immunity, Quillen College of Medicine, East Tennessee State University, Johnson City, Tennessee, USA; University of Texas MD Anderson Cancer Center, Houston, Texas, USA

**Keywords:** *Candida albicans*, caspofungin adaptation, cell wall remodeling, 1,3-*β*-glucan exposure, chromosome 5 genes

## Abstract

*Candida albicans* is part of normal microbiota; however, it can cause superficial and life-threatening infections in immune-compromised individuals. As the use of drugs from the echinocandin (ECN) class for the treatment of candidiasis is increasing, resistance against ECNs is also emerging. We identified 10 genes on *C. albicans* chromosome 5 (Ch5) that are simultaneously downregulated to decrease susceptibility to ECN caspofungin. Independent knock-out experiments demonstrated that these genes act overall to decrease the 1,3-*β*-glucan level in the cell wall, change levels of mannan and chitin, and affect expression of *FKS* genes. Importantly, 10 genes on Ch5 increase or decrease surface exposure of the immunogenic epitope 1,3-*β*-glucan. Our data indicate functions for previously uncharacterized orf19.970 and orf19.4149.1, of which orf19.970 has no human ortholog. Our data also indicate new functions for the genes *DUS4*, *RPS25B*, *UAP1*, *URA7*, *RPO26*, *HAS1*, and *CKS1*, whereas the function of *CHT2* as a negative regulator of ECN susceptibility has been previously established. Importantly, half of the above genes are essential; hence, indispensable processes are involved in the adaptation to ECNs. Our results suggest that a novel group of genes works in concert to control adaptation to ECNs and point to potential new drug targets.

**IMPORTANCE:**

*Candida* infections are often fatal in immuno-compromised individuals, resulting in many thousands of deaths per year. Caspofungin has proven to be an excellent anti-*Candida* drug and is now the frontline treatment for infections. However, as expected, the number of resistant cases is increasing; therefore, new treatment modalities are needed. We are determining metabolic pathways leading to decreased drug susceptibility in order to identify mechanisms facilitating evolution of clinical resistance. This study expands the understanding of genes that modulate drug susceptibility and reveals new targets for the development of novel antifungal drugs.

## INTRODUCTION

*Candida albicans* is an opportunistic unicellular fungus that lives as normal microbiota in human gut and genital organs (1, 2). Candidiasis caused by *C. albicans* along with other non-albicans species is emerging as a major problem worldwide (3, 4). As echinocandins (ECNs) are being used as frontline treatment against candidiasis, there is a slow, but consistent increase in ECN resistance in *Candida* species (1, 4). The only known mechanisms of clinical resistance to ECNs in *C. albicans* are point mutations in *FKS1* (orf19.2929) which encodes for the catalytic subunit of the 1,3-*β*-glucan synthase complex (5). These mutations cause dramatic elevation of *C*. *albicans* minimum inhibitory concentration (MIC) values to ECNs and reduce the sensitivity of the glucan synthase to up to 3,000-fold, the concentration of ECN caspofungin (CAS) inhibiting 50% of enzymatic activity (IC50) (6). However, there are mechanisms that can decrease susceptibility to ECNs without any mutations in the *FKS1* gene. These mechanisms confer a less dramatic decrease of drug susceptibility and do not confer clinical resistance. As shown earlier, either trisomy of *C. albicans* chromosome 2 (Ch2) or monosomy of Ch5 confers a decrease of susceptibility to CAS reviewed in reference (7). It was also shown by various laboratories that there are a number of genes in *C. albicans* genome that can modulate ECN susceptibility reviewed in reference (8). Furthermore, we have previously generated *C. albicans* mutants with decreased susceptibility to CAS in the absence of *FKS1* mutations by direct exposure of *C. albicans* cells to CAS on Petri dishes in independent parallel experiments (9). [Here, we call such mutants with decreased ECN susceptibilities “adapted” or “tolerant.” See also reference (10) for more details of these mutant ECN adaptation profiles to CAS, anidulafungin (ANI) and micafungin (MFG) and associated Minimum Inhibitory Concentrations (MICs).] We found in a similar study of *C. glabrata* by another laboratory that adaptation of *C. albicans* to cidal ECNs involves robust genome-wide changes at the transcription level involving hundreds and even thousands of genes (8, 11). The complexity of the adaptation process was recently further elucidated by demonstrating that CAS-adapted mutants can acquire a limited number of unique DNA changes genome wide, predominantly single-nucleotide substitutions, half of which occurred as hotspots and is shared among the mutants (12).

This study explores the network of genes through which *C. albicans* adapts to ECN drugs. We have started the identification of groups of genes whose expression changed in the same direction across independent CAS-adapted mutants. We characterized five simultaneously upregulated genes from Ch2 that are important for the cell wall structure and positively control CAS and ANI susceptibility (8). Here, we continue the identification and characterization of genes with similar expression changes in CAS-adapted mutants of independent origins. We have analyzed 22 genes residing on Ch5 that are simultaneously downregulated from Ch5 in concert with the cohort of upregulated genes from Ch2. We subsequently focused on a subset of 10 genes and demonstrated that they serve as negative regulators of ECN susceptibility and act, overall, to diminish the amount of immunogenic epitope *β*-glucan in the cell wall and to change the levels of two other major cell wall components, mannan and chitin. These 10 genes either down- or upregulate three *FKS* genes (13) of which *FKS1* is the site of clinical resistance (see above). Finally, and most important, the 10 Ch5 genes split into two sub-groups to control *β*-glucan surface exposure in an opposite fashion, some genes masking while the other unmasking *β*-glucan.

## RESULTS

### Identification and validation of 10 genes on Ch5 serving as negative regulators of susceptibility to ECNs

We have continued our search for the groups of genes that have a role in *C. albicans* adaptation to CAS and other ECNs.

**Fig 1:**
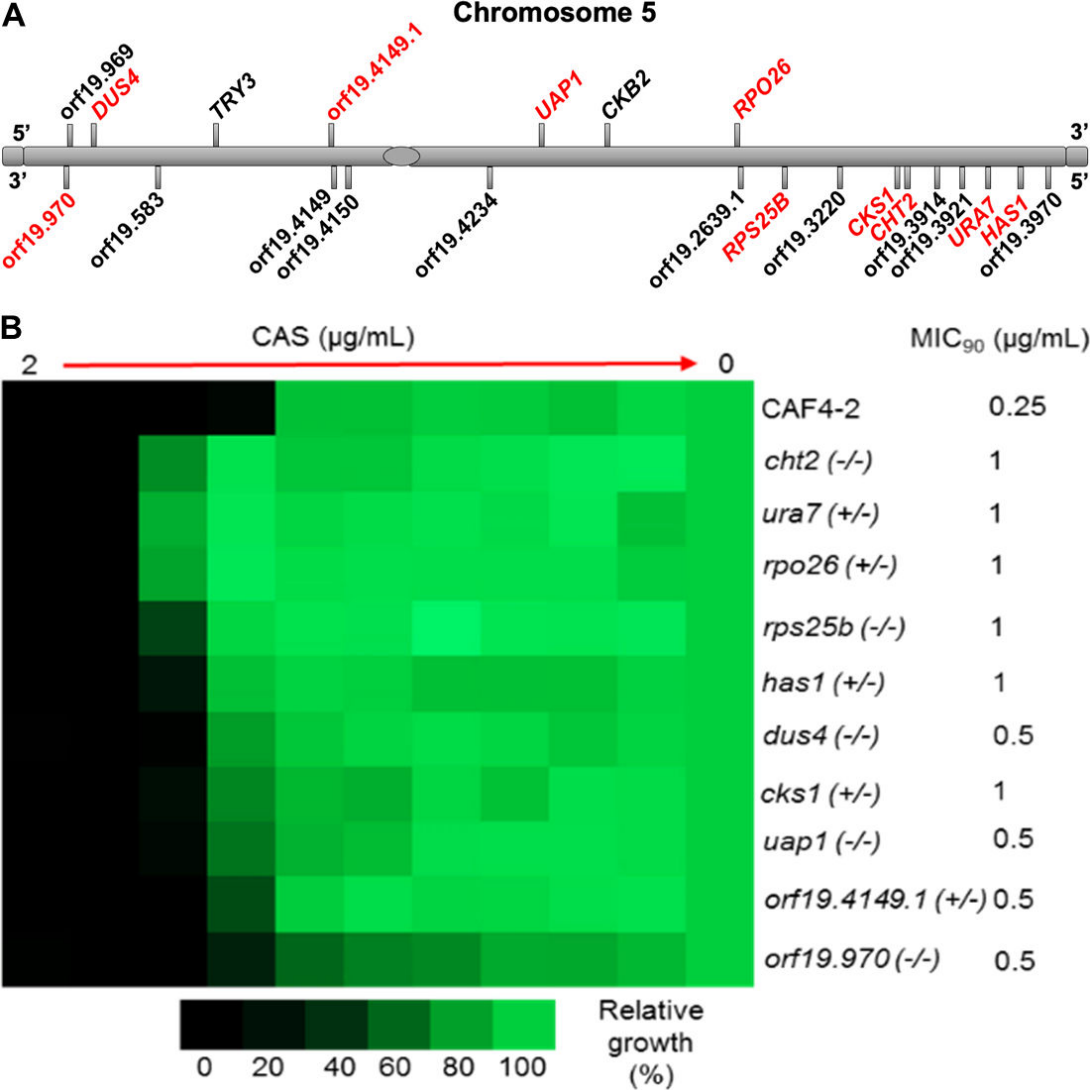
Validation of 10 KO mutants on Ch5 with broth microdilution assay vs parental CAF4-2. (**A**) A cartoon presenting the distribution of 22 downregulated genes across Ch5 (not to scale). (**B**) The heat map shows the increased growth of Ch5 KO mutants vs their parental CAF4-2, as expected. CAS refers to caspofungin. Shown are the parental strain CAF4-2 and 10 independent mutants lacking both copies of *CHT2*, *DUS4*, *RPS25B*, *UAP1*, or orf19.970, as well as one copy of essential *URA7*, *RPO26*, *HAS1*, *CKS1*, or orf19.4149.1. Names, genotypes of strains, and MIC_90_ are indicated on the right. The assay was conducted according to CLSI (Clinical and Laboratory Standards Institute) methods in RPMI 1640 medium with 2% glucose. The assay included a maximum caspofungin concentration of 2 µg/mL and twofold serial dilutions. A total of 10^3^ cells were inoculated into each well in four technical replicates, and the tray was incubated at 35°C for 48 h. Control wells without the drug or without cells were included. The no-cell control was used to subtract the background. The no-drug control was used for normalization. The color bar for percent growth is presented below the heap map.

We identified 22 genes residing on Ch5 (Fig. 1A) that are downregulated in concert across CAS-adapted mutants according to our bioinformatic analysis (8). Downregulation of genes in adapted mutants implies that they encode negative regulators of ECN susceptibility. We independently deleted each of 22 genes in the CAF4-2 background. The knockout (KO) rates of growth vs parental CAF4-2 are presented in Fig. S1. We then tested the KOs for their growth in the presence of each ECN: CAS, ANI, or MFG with broth microdilution assay. Ten KOs lacking both copies of *CHT2* (orf19.3895), *DUS4* (orf19.9660), *RPS25B* (orf19.6663), *UAP1* (orf19.4265), orf19.970, or a single copy of essential *URA7* (orf19.3941), *RPO26* (orf19.2634), *HAS1* (orf19.3962), *CKS1* (orf19.1282), and orf19.4149.1 showed, as expected for deletion of negative regulators, increased growth vs parental CAF4-2 in the presence of CAS (Fig. 1B). CAS MIC at 90% inhibition of growth (MIC_90_) of the KOs increase approximately two- to fourfold. Similar tests in the presence of ANI showed approximately a twofold increase of MIC_90_ for all, but two KOs (Fig. S2), while only two KOs showed approximately a twofold increase of MIC_90_ in the presence of MFG (Fig. S2). Overall, broth microdilution experiments with ANI and MFG support the experiment with CAS (Fig. 1B), and altogether, the above tests validate the role of 10 genes in negative control of ECN susceptibilities. On a special note, the role of *CHT2* as a negative regulator of ECN susceptibility has been previously reported (14).

The remaining 12 KOs: *ckb2* (orf19.4297), *try3* (orf19.1971), orf19.4234, orf19.3220, orf19.969, orf19.3970, orf19.3921, orf19.4149, orf19.2639.1, orf19.4150, orf19.3914, and orf19.583, showed no role in CAS susceptibility when tested with broth microdilution assay in the presence of caspofungin (Fig. S3). Strikingly, 8 out of 12 corresponding genes have no known function. Four out of 12 genes (orf19.969, orf19.3970, orf19.4149, and orf19.2639.1) are essential; 3 out of 12 genes (orf19.3970, orf19.3921, and orf19.4149) have no human orthologs, and one gene (orf19.969) is implicated in fungal-type cell wall organization. In order to expand the study of the function of these 12 KOs, we then conducted an alternative spot assay with one of each CAS or ANI. We found that three KOs (orf19.4234, orf19.969, and orf19.583) showed better growth as compared with parental CAF4-2 in the presence of ANI (Fig. S4). This result was supported by better growth of these KOs in the presence of CAS in the two last spots on the right (Fig. S4). Of the remaining eight KOs, some showed similar or less growth vs parental CAF4-2.

One gene out of 12, *CKB2* (orf19.4297) showed no growth at all with either CAS or ANI spot assay (Fig. S4). *CKB2* encodes the *β*′ regulatory subunit of *C. albicans* Ser/Thr protein kinase CK2 (casein kinase II), an important, highly conserved eukaryotic protein, which is well studied in model yeast *Saccharomyces cerevisiae* (Saccharomyces Genome Database). CK2 is known to phosphorylate hundreds of substrates, to control several signaling pathways, and to be an emerging target in various human diseases (15). Importantly, deletion of *CKB2* renders hypersensitivity to ECNs in pathogenic *C. albicans* but not in the model yeasts *S. cerevisiae* and *Schizosaccharomyces pombe* (16). The lack of growth of the *ckb2* KO in spot assays confirms the original observation of CK2 implication with ECN susceptibility. Apparently, *CKB2* can be downregulated in the CAS-adapted cells, as determined by the bioinformatic analysis of RNA-seq data (8). We speculate that, by the analogy with *CKB2*, the remaining 11 out of 12 genes are of special importance for the cell. This might explain why their KOs display a range of growth levels vs parental CAF4-2 from an expected increase to unexpected reduction (Fig. S4). We tentatively considered all 12 genes as negative regulators of ECN susceptibility. However, further study is needed, employing, in particular, combined deletion of the genes, in order to formally validate the genes. In this study, we will focus on 10 genes (*CHT2*, *DUS4*, *RPS25B*, *UAP1*, orf19.970, *URA7*, *RPO26*, *HAS1*, *CKS1*, and orf19.4149.1) that showed a clear increase in MICs with broth microdilution assay (Fig. 1B).

### Ten Ch5 genes encoding negative regulators of ECN susceptibility control the cell wall

**Fig 2:**
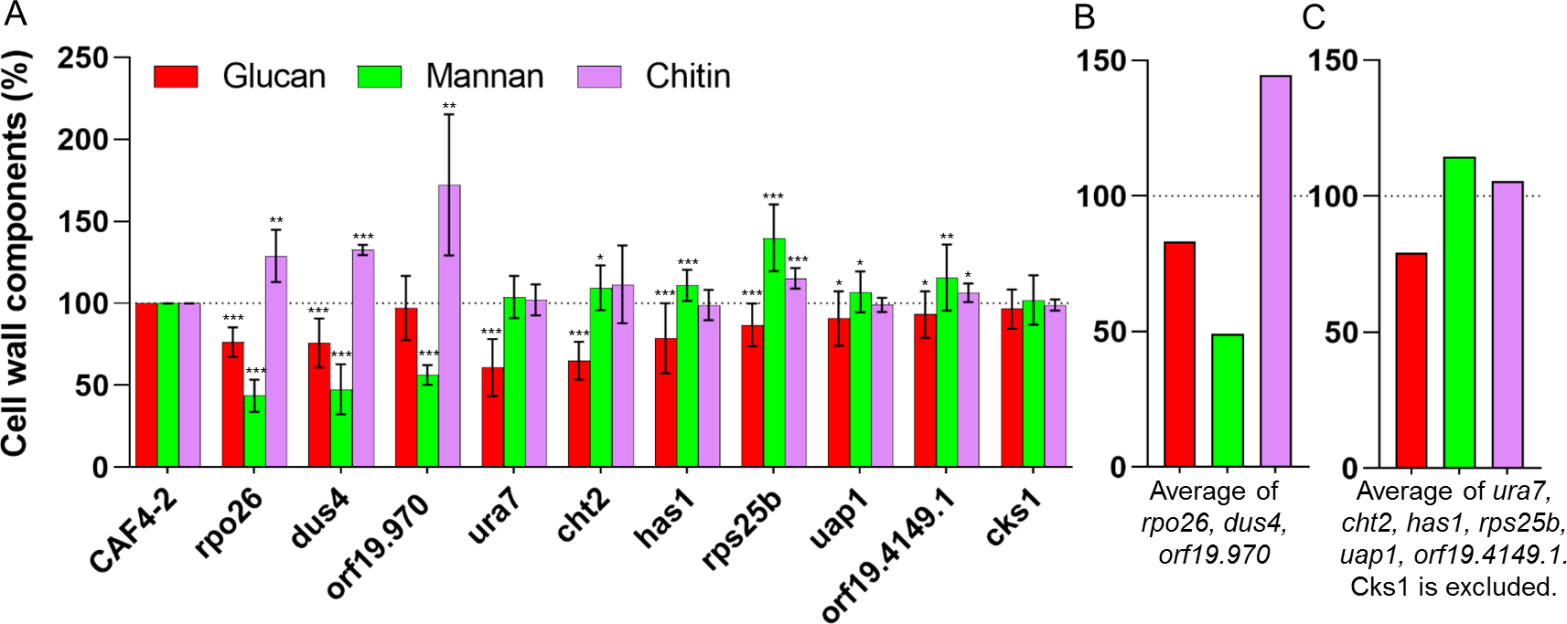
Changes of cell wall mannan, glucan, and chitin in 10 KOs from Ch5. (**A**) Parental strain CAF4-2 and five independent KOs lacking both copies of *CHT2*, *DUS4*, *RPS25B*, *UAP1*, or orf19.970, and five independent KOs lacking one copy of *URA7*, *RPO26*, *HAS1*, *CKS1*, or orf19.4149.1. Measurements were performed on at least two biological replicates, each replicates with four technical replicates. The amount of glucan, mannan, and chitin in the parental strain is set as 100%. The asterisks indicate a *P* value of <0.05 (*), <0.01 (**), or <0.001 (***), as determined using Student’s *t*-test. (**B** and **C**) Cartoons presenting two different patterns of glucan, mannan, and chitin, as averaged for two groups of genes as indicated.

We asked whether 10 genes for negative regulation of ECN susceptibility affect the cell wall. We determined the amounts of three major components of the cell wall (glucan, mannan, and chitin) in each KO. As presented in Fig. 2A, a decrease of glucan could be found in all KOs, but two, the essential *CKS1* gene, of which only one copy could be removed and orf19.970. This suggests that simultaneous downregulation of at least eight genes on Ch5 in cells serves to diminish the amount of cell wall glucan.

In two KOs (*rpo26* and *dus4*), a noted decrease in glucan is combined with a dramatic decrease of mannan and an increase of chitin, as summarized in Fig. 2B, while orf19.970 also showed a dramatic decrease of mannan and an increase of chitin, albeit no significant decrease of glucan. In contrast, in the remaining KOs, mannan does not change or is increased, as summarized in Fig. 2C, while chitin does not change or is increased (Fig. 2A and C).

**Fig 3:**
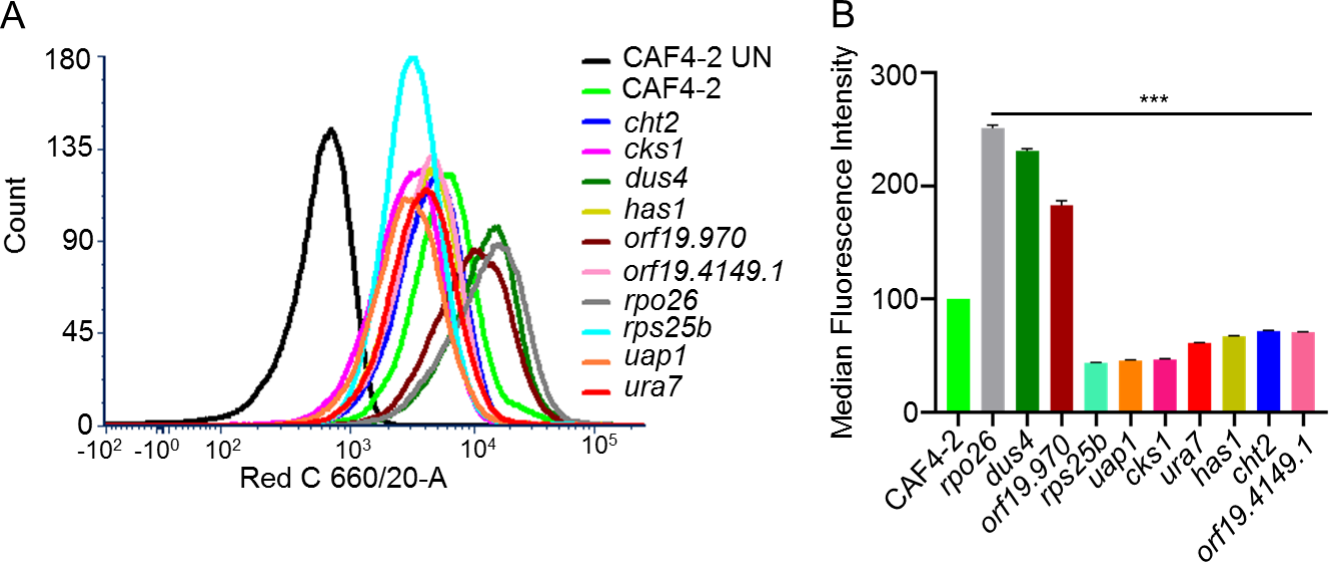
Increased or decreased glucan exposure in 10 Ch5 KOs vs parental CAF4-2. Glucan exposure was measured with FACS using hDectin-1a and anti-IgG antibody conjugated with Alexa Fluor 647. (**A**) Representative histogram showing at least 10^4^ singlet population of Ch5 KO mutants in red C channel. CAF4-2 UN stands for unstained control. Note that peaks of three outstanding KO mutants *rpo26*, *dus4*, and orf19.970 shifted to the right indicating increased fluorescence as compared with parental CAF4-2. While peaks of remaining KO mutants *rps25b*, *cht2*, *cks1*, *has1*, orf19.4149.1, *ura7*, and *uap1* shifted to the left indicating decreased fluorescence. (**B**) Bar graph presentation of normalized median fluorescence intensity of each KO from three biological repeats that include nine technical repeats. The amount of glucan exposure was calculated relative to that in the parental strain CAF4-2, which was considered 100%. The asterisks indicate a *P* value of <0.001 (***), as determined using Student’s *t*-test.

In order to detail the differences in the cell wall remodeling in two groups of KOs, we performed fluorescence-activated cell sorting (FACS) assay with hDectin-1a. The *rpo26*, *dus4*, and orf19.970 KOs display dramatically decreased mannan and increased exposure of glucan, as compared with the parental CAF4-2 (Fig. 3). Hence, *RPO26*, *DUS4*, and orf19.970 act in cells to mask *β*-glucan. In contrast, the remaining KOs (*cht2*, *rps25B*, *uap1*, *ura7*, *has1*, *cks1*, and orf19.4149.1) showed decreased glucan exposure. Therefore, *CHT2*, *RPS25B*, *UAP1*, *URA7*, *HAS1*, *CKS1*, and orf19.4149.1 act in cells to unmask *β*-glucan. Thus, our data indicate multiple mechanisms by which negative regulators from Ch5 govern the cell wall.

### A variety of phenotypes are observed with 10 Ch5 genes encoding negative regulators of ECN susceptibility

**Fig 4:**
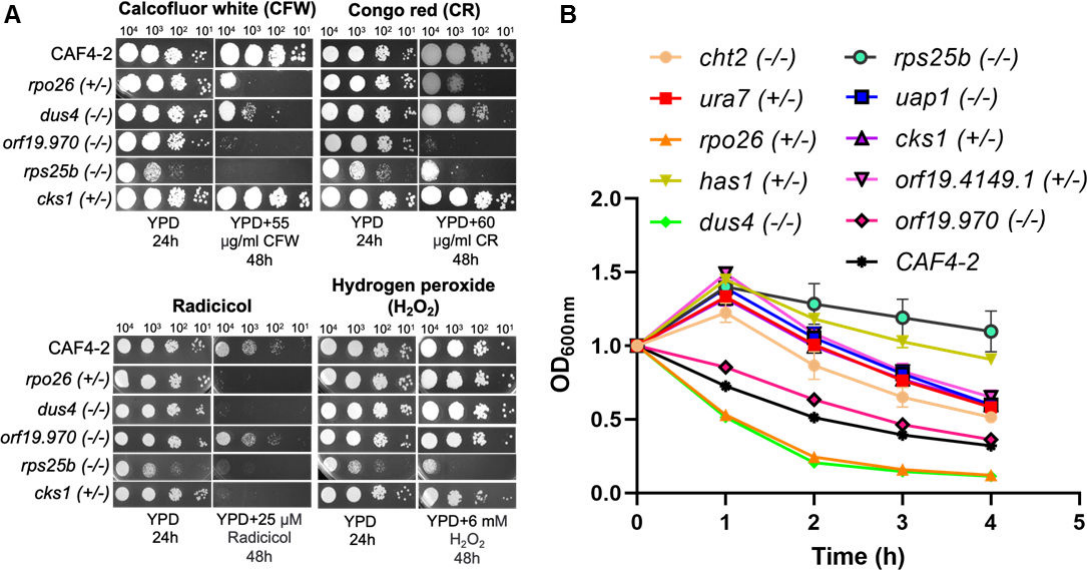
Various phenotypes of 10 KOs from Ch5 vs parental strain CAF4-2. (**A**) Spot assay shows growth of five KO mutants lacking both copies of *DUS4*, orf19.970, *RPS25B*, or one copy of essential *RPO26* and *CKS1* vs parental CAF4-2 on yeast extract-peptone-dextrose (YPD) medium supplemented with calcofluor white, congo red, radicicol, or hydrogen peroxide, as indicated. Strains, genotypes, cell number spotted and incubation time of plates, and control growth on YPD medium are shown. Note that the five remaining KOs showed no growth defect by these assays. (**B**) Survival in the presence of zymolyase of 10 KO mutants lacking both copies of *CHT2*, *DUS4*, *RPS25B*, *UAP1*, and orf19.970 or one copy of essential *URA7*, *RPO26*, *HAS1*, *CKS1*, and *orf19.4149.1* vs parental CAF4-2. The means and standard deviations of the optical density (O.D_600nm_) values of each strain from four technical replicates are plotted against the time of incubation with zymolyase. The O.D_600nm_ at time point zero is scaled to 1 for better representation. Student’s *t*-test was performed between the parental strain and each mutant strain at the 4-h time points. *P* values for the time point ranged from 0.001055 to 1.77 × 10^−7^, except for orf19.970 where the *P* value was 0.076.

In order to better understand the functions of 10 Ch5 genes, we conducted spot assays to test the survival of corresponding KO mutants in the presence of cell wall stressors calcofluor white (CFW) or congo red (CR) and the cell membrane disruptor SDS as well as in the presence of chemical agents affecting various metabolic or signaling pathways, including tunicamycin for endoplasmic reticulum stress, geldanamycin, and radicicol for the inhibition of Hsp90, cyclosporin A or FK506 for the calcineurin pathway, H_2_O_2_ for oxidative stress, aureobasidin A for sphingolipid biosynthesis, hygromycin B for protein synthesis, and caffeine for antifungal activity. As presented in Fig. 4A; Table 1, three KOs (*rpo26*, *dus4*, and orf19.970), having increased exposure of glucan (see above), showed different degrees of increased sensitivity to calcofluor white and congo red that is indicative of their disturbed cell wall. Two of three KOs (*rpo26* and *dus4*) showed inhibition of growth in the presence of radicicol, indicative of *RPO26* and *DUS4* involvement in the *HSP90* pathway. In addition, the *rps25b* KO showed increased sensitivity to calcofluor white and congo red (disturbed cell wall), radicicol (involvement in the *HSP90* pathway), and H_2_O_2_ (involvement in oxidative stress), while growth of the *cks1* KO was inhibited by radicicol (involvement in the *HSP90* pathway).

**TABLE 1:** The summary of phenotypes^*b*^ of 10 deletion strains each lacking one Ch5 gene, as compared with parental CAF4-2^*a*^

Strain, gene ploidy	CFW	CR	Radicicol	H_2_O_2_	Expression of *FKS* genes			Glucan	Glucan exposure	Mannan	Chitin	Zymolyase
					FKS1	FKS2	FKS3					
Parental CAF4-2	0^*c*^	0	0	0	0	0	0	0	0	0	0	0
*RPO26*, +/−	−	−	−	0	−	−	−	−	+	−	+	−
*DUS4*, −/−	−	0	−	0	0	0	+	−	+	−	+	−
orf19.970, −/−	−	−	0	0	−	−	−	0	+	−	+	0
*RPS25B*, −/−	−	−	−	−	+	+	+	−	−	+	+	+
*CKS1*, +/−	0	0	−	0	−	−	−	0	−	0	0	+
*CHT2*, −/−	0	0	0	0	−	−	−	−	−	+	0	+
*UAP1*, −/−	0	0	0	0	−	0	−	−	−	+	0	+
*URA7*, +/−	0	0	0	0	−	−	−	−	−	0	0	+
*HAS1*, +/−	0	0	0	0	−	−	0	−	−	+	0	+
orf19.4149.1, +/−	0	0	0	0	−	−	+	−	−	+	+	+

^*a*^ Shown are growth in the presence of cell wall stressors calcofluor white and congo red in KOs, growth in the presence of Hsp90p inhibitor radicicol and oxidative stress inducer hydrogen peroxide (H_2_O_2_) in KOs, relative expression of *FKS* genes in KOs, level of glucan in cell wall and glucan cell surface exposure in KOs, and mannan and chitin levels in cell wall and resistance to zymolyase.

^*b*^ For the ECN phenotypes see Fig. 2.

^*c*^ 0 stands for the parental level of the phenotype of interest. + or – stands for more or less growth, increase or decrease of expression, increase or decrease of amount or surface exposure, and increase or decrease of resistance, correspondingly.

In addition, we tested for growth of KOs in the presence of zymolyase, an enzyme mixture that lyses the *C. albicans* cell wall (17). Three KOs (*rpo26*, *dus4*, and orf19.970), having increased exposure of glucan, were lysed either significantly faster (*rpo26* and *dus4*) or similarly to the parental strain (orf19.970) (Fig. 4B). This result is consistent with the essential activities of zymolyase that include *β*−1,3-glucan laminaripentao-hydrolase activity and *β*−1,3-glucanase activity (17). We also observed that the remaining KOs that have decreased the exposure of glucan were lysed by zymolyase at a significantly slower rate than parental CAF4-2 (Fig. 4B).

### Ten Ch5 genes encoding negative regulators of ECN susceptibility influence expression of *FKS* genes

**Fig 5:**
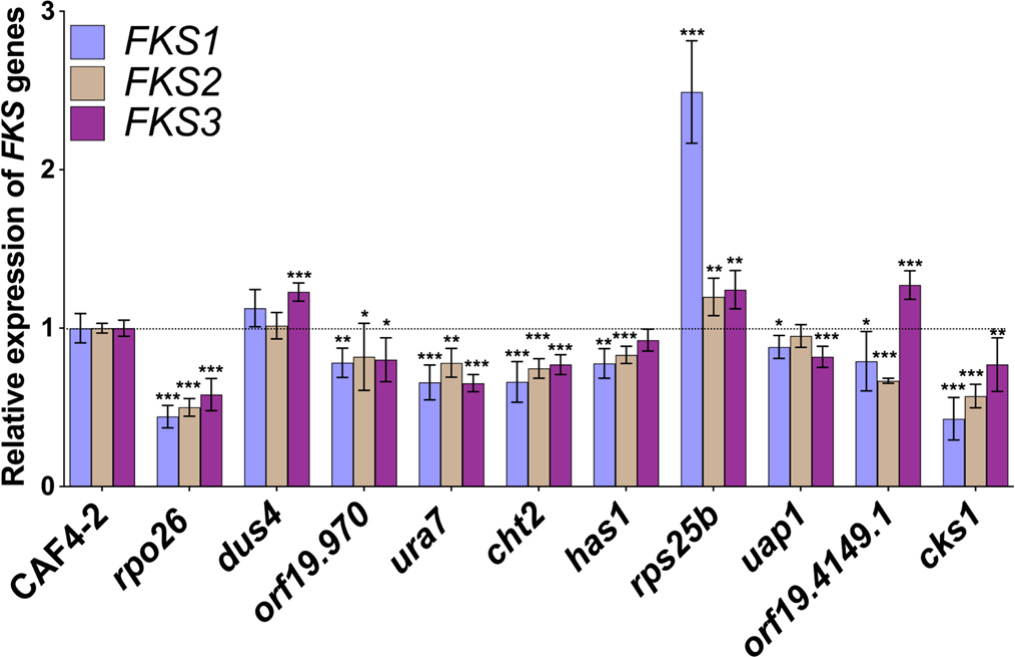
Expression changes of *FKS1*, *FKS2*, and *FKS3* genes in 10 Ch5 KOs vs parental CAF4-2. (**A**) The name of the mutants and relative gene expressions are indicated at the *x-* and *y*-axis, respectively. The qPCR measurements were performed on at least two biological replicates, each replicates with three technical replicates. The asterisks indicate a *P* value of <0.05 (*), <0.01 (**), or <0.001 (***), as determined using Student’s *t*-test. Bars indicate standard deviation.

We asked whether *FKS1*, encoding the catalytic subunit of glucan synthase and also a site of clinical resistance (18), as well as *FKS2* and *FKS3*, which negatively control *FKS1* (13), is affected by the disturbance of 10 validated Ch5 genes. We conducted real-time polymerase chain reaction (RT-PCR) with 10 KOs and found that the *FKS* genes were downregulated in the majority of KOs (Fig. 5). Expression of *FKS2* did not change in the *uap1* KO, while expression of *FKS1* and *FKS2* did not change in the *dus4* KO. These results strongly imply that 10 genes on Ch5 encode negative regulators of ECN susceptibilities that influence *FKS* gene expressions.

### Diverse properties of 10 proteins acting as negative regulators of ECN susceptibility

In order to understand the molecular function of proteins that are encoded by 10 simultaneously downregulated genes on Ch5, we explored the *Candida* Genome Database (CGD) for available information (http://www.candidagenome.org/). The size of proteins ranges from 102 aa (amino acid) to 583 aa. A total of five proteins (Ura7p, Rpo26p, Has1p, Cks1p, and orf19.4149.1p) are essential. One protein orf19.970p has no human ortholog. *URA7* is 44% identical to *Saccharolobus solfataricus* cytidine triphosphate (CTP) synthase. *RPO26* encodes for a subunit of RNA polymerase and is 67% identical to *S. cerevisiae* Rpo26p. *HAS1* is a functional homolog of *S. cerevisiae HAS1*, which encodes for a nucleolar protein of the DEAD-box-ATP-dependent RNA helicase and is involved in ribosome biogenesis. *DUS4* is involved in tRNA-dihydrouridine synthesis and shares 29% identity with the human homolog of tRNA-dihydrouridine synthase catalytic domain. Rps25B is a ribosomal protein sharing 85% identity with *S. cerevisiae* Rps25Bp, which serves as a structure component of ribosome. *UAP1* encodes for UDP-N-acetylglucosamine pyrophosphorylase, which catalyzes synthesis of UDP-N-acetylglucosamine. *CKS1* shares 86% identity with *S. cerevisiae* cell cycle regulatory protein Cks1p. The molecular function of Cks1p is as a cyclin-dependent protein serine/threonine kinase activator. Orf19.4149.1 encodes for a protein component of a small (40S) ribosomal subunit which is identical to 40S-eif1a from *Kluyveromyces lactis*. The putative function of orf19.970 is poorly understood, except that it has a role in microtubule-related processes and shares 30% sequence identity with *S. cerevisiae* BER1.

We could not find any common motifs or domains present when we did multiple protein sequence alignments of 10 genes. This information expands the function of eight previously annotated genes and suggests a function for two non-annotated genes.

### Promoters of 10 Ch5 genes encoding negative regulators of ECN susceptibility contain multiple binding sites for putative transcription factors

**Fig 6:**
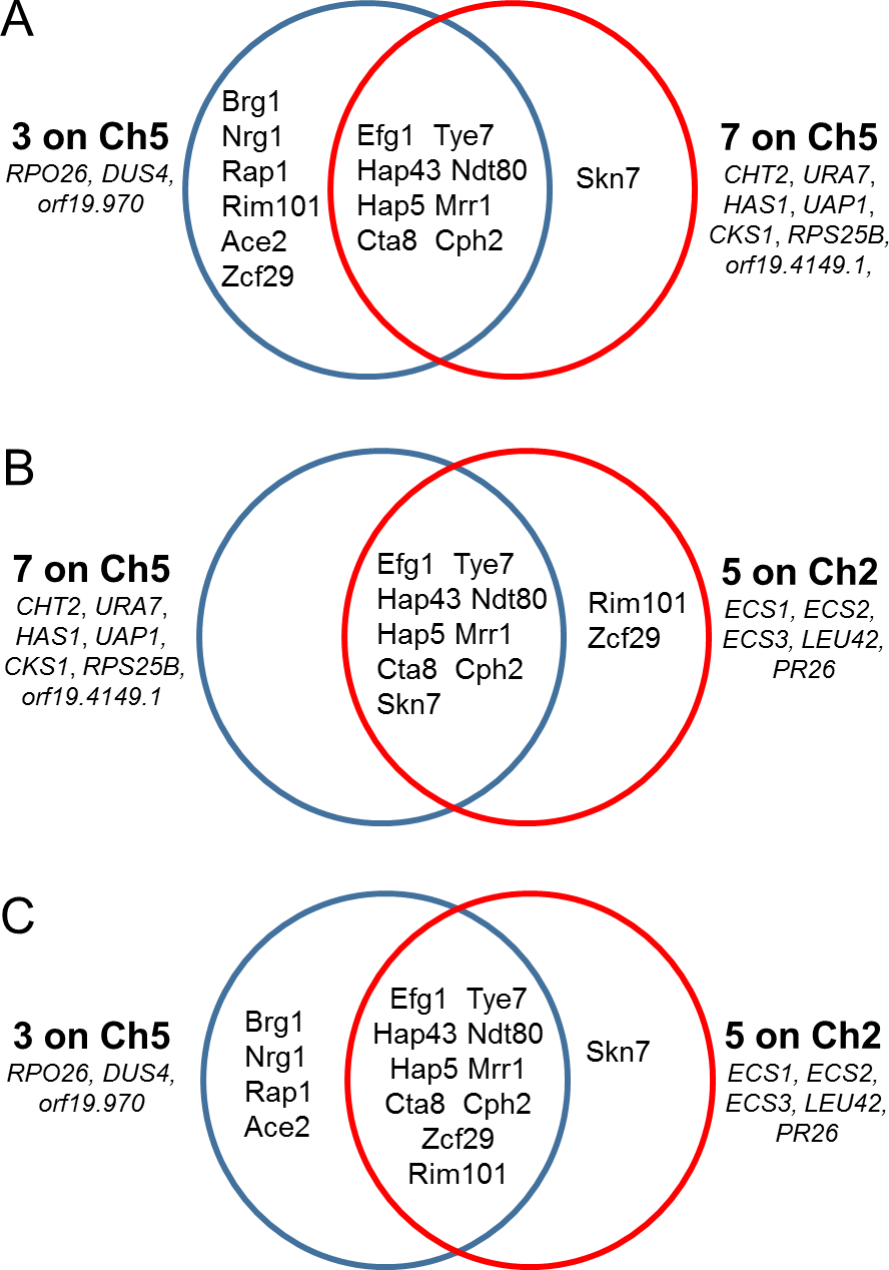
Venn diagrams showing binding sites of TFs within 1 kb upstream sequence of each ORF in two groups of downregulated genes on Ch5 and in a group of upregulated genes on Ch2. Gene names and groups of genes are indicated. Names in intersections of the circles are of corresponding TFs, whose binding sites are shared between the groups of genes. Names in the larger parts of the circles are of corresponding TFs, whose binding sites are shared within the group. (**A**) Three genes on Ch5 having increased glucan exposure vs seven genes on Ch5 having decreased glucan exposure. (**B**) Seven genes on Ch5 having decreased glucan exposure vs five genes on Ch2. (**C**) Three genes on Ch5 having increased glucan exposure vs five genes on Ch2.

In order to get more insight into the simultaneous downregulation of Ch5 genes, we explored 1 kb of the upstream sequence of each open reading frame (ORF) for the presence of consensus transcription factor (TF) binding sites and identified multiple binding sites across the 10 genes. We next compared 3 out of 10 genes with more exposed glucan against 7 genes with less exposed glucan (Fig. 6A). Because 10 downregulated Ch5 genes act in concert with 5 previously described upregulated Ch2 genes (*ECS1*, *ECS2*, *ECS3*, *LEU42*, and *PR26*) (8), we also investigated 5 genes on Ch2 for TF binding sites and identified multiple binding sites across the 5 genes. We also compared five Ch2 genes with three and seven Ch5 genes (Fig. 6B and C, respectively).

In all three comparisons, we found they collectively share binding sites between the groups of genes for the following TFs: Efg1, Tye7, Hap43, Ndt80, Hap5, Mrr1, Cta8, Cph2, Skn7, Zcf29, and Rim101 (see the intersections of the circle of Venn diagrams in Fig. 6A through C). To assess how frequently these binding sites appear in promoters of genes in the *C. albicans* genome, we analyzed a representative set of 104 promoters. For this purpose, we analyzed 1 kb upstream sequence of 13 randomly chosen genes from each of 8 chromosomes of *C. albicans*. In this set of 104 sequences, we found that Cph2, Mrr1, Hap5, and Cta8 were present in all sequences. Hence, these binding sites are not specific for the regulation of our genes of interest, while the remaining binding sites were present in the following sequences: Efg1 (69%), Tye7 (94%), Hap43 (93%), Ndt80 (73%), Skn7 (75%), Zcf29 (82%), and Rim101 (68%). Hence, these binding sites appear relatively frequently across the genome and can be hardly important for specific regulation.

We also performed an analysis using representative random 104 upstream sequences for the TFs that are common for each group of genes, as shown in the larger parts of the circles of Venn diagrams in Fig. 6A through C. We found, similarly, relatively high frequencies of TF binding sites, ranging from 52% to 89%, in these randomly chosen promoters. In future studies, the definitive comparative analysis of promoters will include ATAC-seq and ChIP-seq approaches.

## DISCUSSION

Here, we show that 10 genes (*RPO26*, *DUS4*, orf19.970, *RPS25b*, *CHT2*, *CKS1*, *HAS1*, orf19.4149.1, *URA7*, and *UAP1*) that reside on Ch5 and are downregulated in concert in CAS-adapted mutants (8) negatively control ECN susceptibility. All but two of these genes act to decrease the amount of immunogenic epitope *β*-glucan of the cell wall. This overall action of the Ch5 genes is the same as the previously reported action of a cohort of five genes on Ch2 that are upregulated in concert, and also in concert with Ch5 genes, to decrease the amount of *β*-glucan (8). Taken together, these results indicate that *C. albicans* possesses a complex regulatory system, which simultaneously up- and downregulates genes to decrease the level of the cell wall *β*-glucan in CAS-adapted mutants. Such decrease is reminiscent of earlier studies demonstrating that temporary exposure of *Candida* cells to CAS induces a decrease of *β*-glucan in the cell wall (19).

The simultaneous regulation of two cohorts on Ch2 and Ch5 needs further study. However, shared binding sites for Efg1, Tye7, Hap43, Ndt80, Hap5, Mrr1, Cta8, and Cph2 in all sequence comparisons could be indicative of such regulation. Furthermore, Rim101, whose binding site is also shared between five genes on Ch2 and three genes on Ch5, is already known to have a role in ECN tolerance (20).

*β*-Glucan is a major component of pathogen-associated molecular pattern (PAMP) that along with mannan is recognized by the host immune system to combat fungal infection (21). The 10 Ch5 genes split into two subgroups regarding control of *β*-glucan surface exposure, which is also called glucan masking and unmasking (21). Three of the 10 genes (*RPO26*, *DUS4*, and orf19.970) facilitate a decrease of *β*-glucan exposure for cells to evade the host immune system, hence *β*-glucan masking, whereas the remaining seven genes (*RPS25b*, *CHT2*, *CKS1*, *HAS1*, orf19.4149.1, *URA7*, and *UAP1*) facilitate an increase of *β*-glucan exposure, making cells better recognized by the host immune system, hence *β*-glucan unmasking. We propose that balancing between the action of masking and unmasking genes is important to mount an appropriate cell response to the environmental cues. These genes collectively act, in an undefined manner, to control the highly immunogenic cell wall epitope *β*-glucan and are important for the development of therapeutic strategies.

Previous studies from other laboratories found that at least 25 genes: *CHO1*, *KRE1*, *KRE5*, *FGR41*, *CPW419*, *MKC1*, *CHS3*, *PHR2*, *SSN8*, *CEK1*, *ENG1*, *DFL1*, *CRZ1*, *GRP1*, *MNT1*, *MNT2*, *RIM101*, *BCR1*, *GPA2*, *TEK1*, *CYR*, *AOX1*, *GOA1*, *UPC2*, and *SOD1*, as well as *MNN* gene families that reside on various chromosomes, regulate *β*-glucan surface exposure (21 – 36). All these genes, but *KRE1*, were reported to act in the cell to mask *β*-glucan. None of the above genes matches the 10 Ch5 genes that we report here to either mask or unmask *β*-glucan.

As shown by our KO experiments, another importance of these 10 Ch5 genes lies in their ability to impact the expression level of *FKS* genes. We speculate that the 10 Ch5 genes mediate a decrease of the *β*-glucan amount in the cell wall through *FKS* genes.

Pathways to which these 10 Ch5 genes belong remain rather elusive. For example, the 10 Ch5 genes we identified are not involved with Ca^2+^-calcineurin (Cna1) signaling, which is known to govern both *β*-glucan masking and unmasking (26). On the other hand, we find that Hsp90p, of which calcineurin is a client protein (37, 38) and is a conserved molecular chaperone that facilitates the folding and function of hundreds of client proteins reviewed in reference (39), is involved with the regulation of four genes *RPO26* and *DUS4* (masking) and *RPS25b* and *CKS1* (unmasking). We conclude that these four Ch5 genes (*RPO26*, *DUS4*, *RPS25b*, and *CKS1*) are involved in still-to-be-understood pathways governed by Hsp90p. Based on the results from several laboratories including our data, we also conclude that the *C. albicans* genome harbors multiple genes that either mask or unmask *β*-glucan and we propose that a collective action of these genes determines the degree of surface exposure of *β*-glucan.

We also observed that disturbing some genes on Ch5 exerts a significant effect on the mannan and chitin levels. Disturbing genes masking *β*-glucan (*RPO26*, *DUS4*, and orf19.970) is associated with a decreased level of mannan and a rather dramatic increase of chitin. In contrast, disturbing Ch5 genes unmasking *β*-glucan (*RPS25b*, *CHT2*, *CKS1*, *HAS1*, orf19.4149.1, *URA7*, and *UAP1*) is associated with predominantly increased levels or no change of mannan, as well as with a moderate increase, if any, of chitin. Each of these changes was previously recognized as a mechanism controlling *β*-glucan unmasking in CAS-treated cells of *C. albicans* (19, 26, 27, 36). The decrease of glucan due to exposure to CAS is also known to be accompanied by an increase of chitin and mannan and can be viewed as a compensatory means to fortify the cell wall (19, 40). Taken together, these data indicate a complexity of processes remodeling the cell wall in CAS-adapted mutants.

Our data show that genes on Ch5 negatively controlling ECN susceptibilities are involved with more than one mechanism determining the pattern of cell wall remodeling. Importantly, these mechanisms are coupled with either masking or unmasking of *β*-glucan, with the later needing further clarification in future studies.

Our results strongly imply that the 10 Ch5 genes are important for ECN adaptation and for survival in the host. The essentiality of 5 out of 10 genes (*URA7*, *RPO26*, *HAS1*, *CKS1*, and orf19.4149.1) and the involvement of the 10 in the expression of the *FKS* genes that are responsible for *β*-glucan synthesis support this conclusion. Further studies are needed to detail how two cohorts of genes, both positive regulators from Ch2 (8) and negative regulators from Ch5, control adaptation to ECNs by cell wall remodeling. These future studies will identify what genes we choose to focus on for potential antifungal drug development.

## MATERIALS AND METHODS

### Strains, plasmids, and primers

We used *C. albicans* Ura^−^ strain CAF4-2, a derivative of the reference strain SC5314, as a recipient strain to delete genes (14). The primers and plasmids used in this study are presented in Table S1.

### Maintenance and growth of strains and media

Cells were maintained, stored, and grown using our standardized approach that prevents the induction of chromosome instability, as previously described (41). This approach favors maintaining the population of cells that represents a major fraction of cells (42). Briefly, cells were stored at −80°C. When needed, cells from a −80°C stock were streaked for independent colonies onto yeast extract-peptone-dextrose plates and incubated at 37°C until young colonies with a size of approximately 1 × 10^5^ to 3 × 10^5^ cells/colony grew up. Young colonies were collected, a proper dilution in sterile water was prepared with the aid of a hemacytometer, approximately 3,000 colony-forming units (CFU) were plated onto each plate, and plates were incubated until young colonies appeared. Cells were stored in a 25% (vol/vol) glycerol solution at −80°C to interrupt metabolism and routinely grown at 37°C.

YPD medium was prepared using 1% yeast extract, 2% peptone, and 2% dextrose. RPMI 1640 medium (Sigma, St. Louis, MO, USA) was supplemented with 2% glucose. In order to prepare solid medium, 2% (wt/vol) agar was added. Nourseothricin at 150 mg/mL (Jena Bioscience GmbH, Jena, Germany), uridine at 50 mg/mL (Sigma-Aldrich, St. Louis, MO, USA), CAS (Merck Sharp & Dohme Corp., Kenilworth, NJ, USA), or anidulafungin (Pfizer Inc., New York, NY, USA) was added when needed. We also used Zymolyase 100T (U.S. Biological, Swampscott, MA, USA).

### Gene deletions by the transient CRISPR method

We used a transient CRISPR-Cas9 method allowing the deletion of both copies of the target gene in *C. albicans* with a single transformation (43). The *C. albicans* cells were transformed with the Cas9 expression cassette, the sgRNA expression cassette, and the homologous repair templates at a ratio of 1 µg/1 µg/3 µg by lithium acetate transformation method (14). *NAT^R^* transformants were selected on YPD plates supplemented with 150 mg/mL of nourseothricin. The correct target gene deletion was verified by PCR amplification using the 5′-flanking region and either the gene of interest (GI) or *NAT^R^* as well as by the amplification of entire GI or *NAT^R^* using 5′- and 3′-flanking regions (8).

### Broth microdilution assay to determine MICs

We employed a broth microdilution assay according to the CLSI document M27-A3 broth microdilution method for yeasts (44), with some modifications (8). The turbidities were measured with a Spark multimode microplate reader (Tecan, Zurich, Switzerland) at 600 nm. Normalized readings were generated in Microsoft Excel and presented as heat maps.

### Spot assay

Cells from −80°C freezer stocks were streaked on YPD plates and incubated at 37°C until colonies (approximately 1–3 × 10^5^ cells per colony) appeared. Colonies then were collected and suspended in sterile distilled water, colony-forming units were counted with a hemocytometer, and serial 10-fold dilutions of cell suspensions were prepared. The corresponding suspensions were plated at 10^4^, 10^3^, 10^2^, and 10^1^ CFU per spot on plates.

### Zymolyase assay

Cells were streaked from a −80°C stock onto YPD plates for independent colonies and incubated at 37°C for 19 h. Colonies were then collected, and cells were counted with a hemacytometer. The assay was conducted as previously described (8).

### Determination of glucan content in the cell wall

The level of bulk of glucans was determined by an aniline blue assay as previously described (8, 13).

### Determination of chitin content in the cell wall

The amount of chitin was determined by measuring the absorbance of glucosamine released by acid hydrolysis of the purified cell wall as described previously (8, 45).

### Determination of mannan content in the cell wall

Mannan contents were determined with the alcian blue staining method as previously described (8, 46).

### Determination of glucan exposure in the cell wall by FACS

Cells were streaked from a −80°C stock onto YPD plates for independent colonies and incubated at 37°C for 19 h. Cells were then collected and counted with a hemacytometer. 3.5 × 10^6^ cells were taken and washed twice with PBS (phosphate-buffered saline). After washing, cells were stained with 0.5 µg/mL of hDectin-1a (catalog no. fc-hdec1a) (InvivoGen, San Diego, CA, USA) at 25°C for 1 h with shaking. Cells were washed again with 1× PBS twice. Secondary staining of cells was done by incubating 4 mg/mL of goat raised Anti-human IgG antibody (catalog no. A-21445) (Invitrogen, Waltham, MA, USA) conjugated with Alexa Fluor 647 at 25°C for 30 min in the dark with shaking. Cells were washed thrice with PBS before being suspended in 1 mL of PBS for FACS. FACS data collected from LSRII/Fortessa/Symphony A1 (Becton, Dickinson and Company, NJ, USA) were analyzed by software FCS Express 7. FACS data were recorded from nine technical repeats from three biorepeats, and each technical repeat contained of 30,000 events. The singlet population of at least 10,000 cells was selected for further analysis of glucan exposure.

### Analysis of promoters

We extracted 1 kb of upstream DNA sequence from ORFs of interest from CGD. The *C. albicans* PathoYeastract server (http://yeastract-plus.org/pathoyeastract/calbicans/formtfsbindingsites.php) was used to find consensus TF binding sites in the extracted upstream sequences. Output files from the server were analyzed for TF binding sites. Shared binding sites between the groups of genes were identified and presented as a Venn diagram.

### Determination of *FKS* genes’ expression by real-time qPCR

Strains were streaked from −80°C frozen stocks on YPD agar and plates were incubated at 37°C until colony size reaches 1–3 × 10^5^ cells/colony. Colonies were suspended in YPD medium and approximately 1,000 colony-forming units were plated on YPD agar plates. The plates were incubated at 37°C until colony size reaches 1–3 × 10^5^ cells/colony. The colonies were collected and suspended in 1 mL of H_2_O in 1.5-mL microcentrifuge tubes (MCTs), centrifuged at 8,000 rpm for 5 min at 4°C, and washed again with 1 mL of H_2_O to remove any residual media present. The cells were suspended in 1 mL of lysis buffer provided in the RNeasy Kit (Qiagen, MD, USA), and 200 µL of glass beads (0.5 mm) was added. The cells were broken by six alternative cycles of 1 min each of vortex and sample incubation in ice. The samples were centrifuged at 10,000 rpm for 3 min at 4°C, lysates were collected in new MCTs, and the rest of the protocol was performed according to RNeasy Kit instructions. A total of 2 µg of total RNA was treated with DNase I (Thermo Fisher Scientific, MA, USA) followed cDNA preparation using multiscript reverse transcriptase (Applied Biosystems, MA, USA) as per manufacturer’s instruction. The quantitative amplification of cDNA was monitored by incorporation of SYBR green in StepOnePlus (Applied Biosystems, MA, USA) real-time qPCR system using PCR Master Mix (Applied Biosystems, MA, USA). The amplification curves were analyzed using StepOnePlus real-time software (Applied Biosystems, MA, USA). The ΔΔCt method was used for relative quantification of mRNA of target and housekeeping genes (47). The *ACT1* gene was used as housekeeping gene in the analysis (48). The quantification, statistical analysis (Student’s *t*-test) was performed in Excel (Microsoft), and the graph was made using GraphPad Prism.

## Data Availability

All data are available in the main text or supplemental material.

## SUPPLEMENTAL MATERIAL

Fig. S1 spectrum.03295-23-s0001.tif Growth curves of *C. albicans* deletion mutants vs parental CAF4-2.

Supplemental figure legends spectrum.03295-23-s0002.docx Legends for Fig. S1 to S4.

Fig. S2 spectrum.03295-23-s0003.tif Broth microdilution assay of 10 Ch5 KOs against micafungin and anidulafungin.

Fig. S3 spectrum.03295-23-s0004.tif Broth microdilution assay of 12 Ch5 KOs against caspofungin (CAS).

Fig. S4 spectrum.03295-23-s0005.tif Susceptibility of 12 KOs from Ch5 to anidulafungin (ANI) and caspofungin (CAS) as determined with spot assay.

Table S1 spectrum.03295-23-s0006.pdf List of primers and plasmids used in the study.

ASM does not own the copyrights to Supplemental Material that may be linked to, or accessed through, an article. The authors have granted ASM a non-exclusive, world-wide license to publish the Supplemental Material files. Please contact the corresponding author directly for reuse.
