# Supplementary figures and images for: Identification of 10 genes on Candida albicans chromosome 5 that control surface exposure of the immunogenic cell wall epitope β-glucan and cell wall remodeling in caspofungin-adapted mutants
Source: Microbiol Spectr. 2023 Nov 15;11(6):e03295-23. doi: 10.1128/spectrum.03295-23 (PMC10714753; doi:10.1128/spectrum.03295-23)

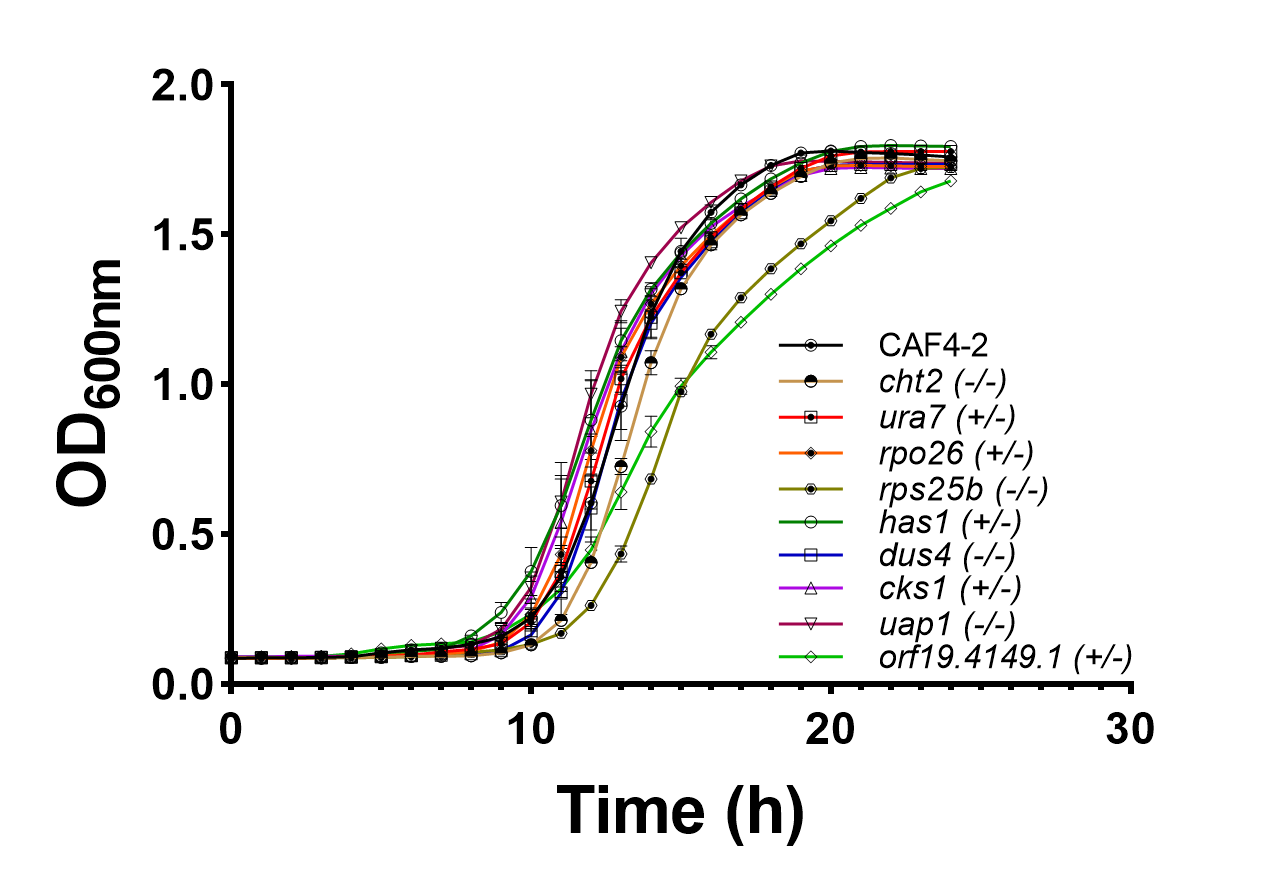

Supplement: Fig. S1 — Growth curves of C. albicans deletion mutants vs parental CAF4-2. [file spectrum.03295-23-s0001.tif]

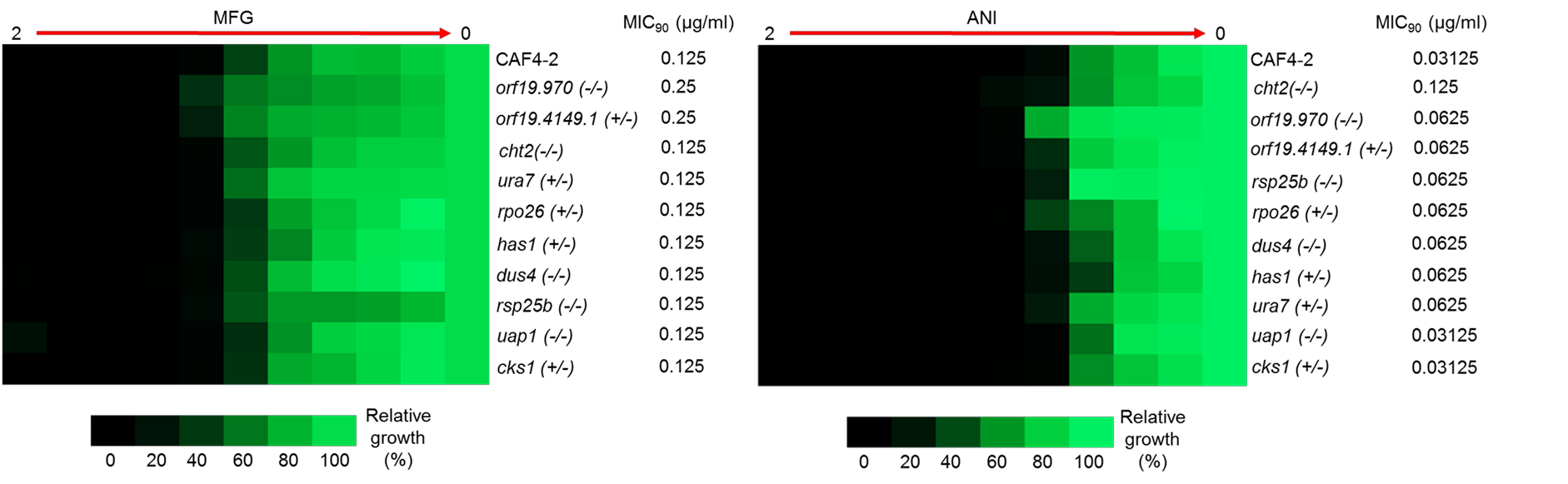

Supplement: Fig. S2 — Broth microdilution assay of 10 Ch5 KOs against micafungin and anidulafungin. [file spectrum.03295-23-s0003.tif]

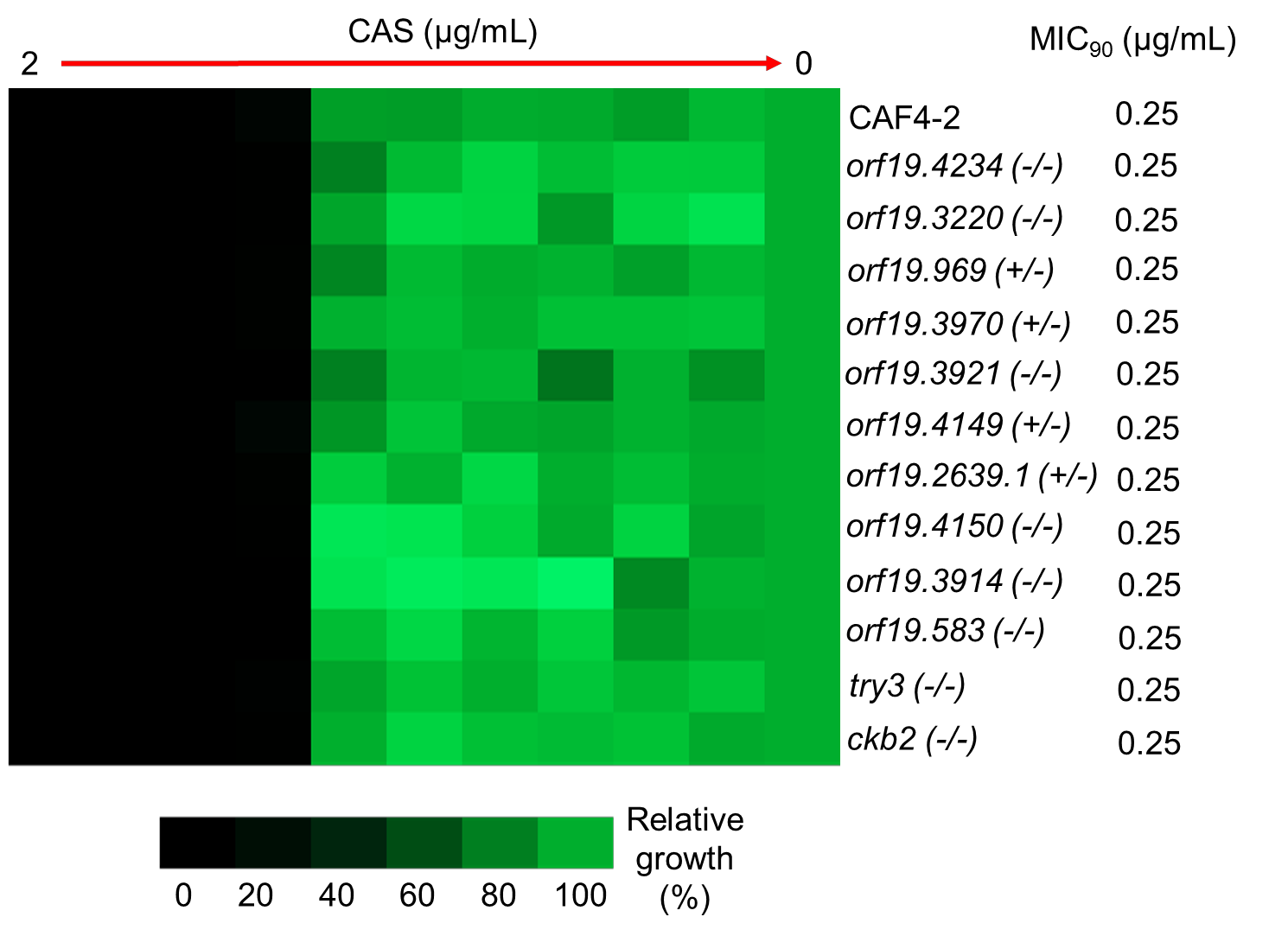

Supplement: Fig. S3 — Broth microdilution assay of 12 Ch5 KOs against caspofungin (CAS). [file spectrum.03295-23-s0004.tif]

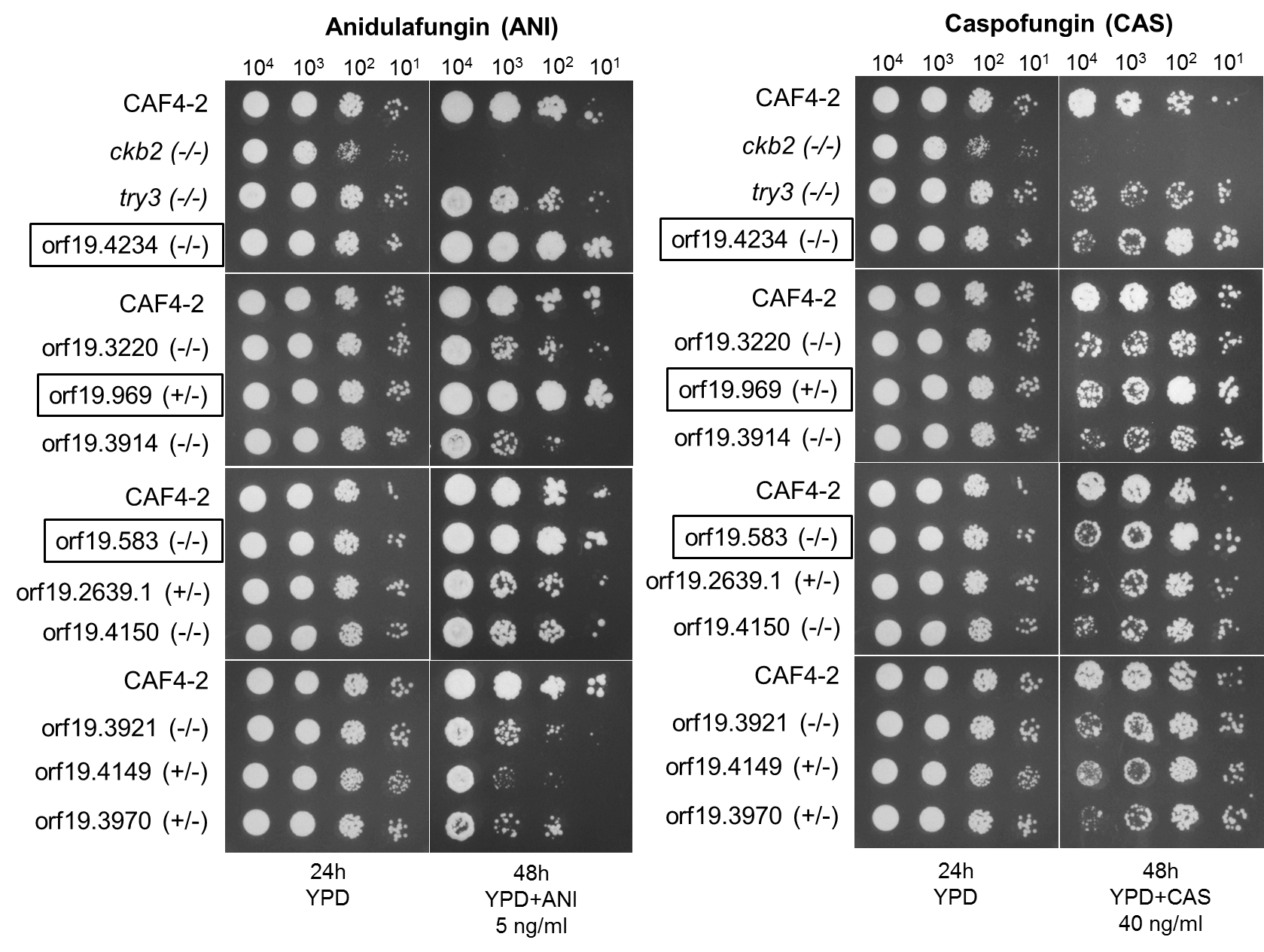

Supplement: Fig. S4 — Susceptibility of 12 KOs from Ch5 to anidulafungin (ANI) and caspofungin (CAS) as determined with spot assay. [file spectrum.03295-23-s0005.tif]
