# Supplementary material for: Identification of 10 genes on Candida albicans chromosome 5 that control surface exposure of the immunogenic cell wall epitope β-glucan and cell wall remodeling in caspofungin-adapted mutants
Source: Microbiol Spectr. 2023 Nov 15;11(6):e03295-23. doi: 10.1128/spectrum.03295-23 (PMC10714753; doi:10.1128/spectrum.03295-23)
Supplement: Supplemental figure legends — Legends for Fig. S1 to S4. [file spectrum.03295-23-s0002.docx]

**SUPPLEMENTRY FIGURE LEGENDS**

**FIG S1**. Growth curves of *C. albicans* deletion mutants vs parental CAF4-2. The panel shows parental strain CAF4-2 and Ch5 independent null mutants lacking *CHT2*, *DUS4*, *RPS25B*, *UAP1* and independent mutants lacking one copy of *URA7*, *RPO26*, *HAS1*, *CKS1* or orf19.4149.1. The cell growth was conducted in YPD medium at 35°C. Optical density was measured at 600 nm and plotted against time. The experiment was performed on three biological replicates with three technical replicates. The mean and standard deviation of optical density shown in the representative data were calculated from one biological replicate.

**FIG S2**. Broth microdilution assay of 10 Ch5 KOs against micafungin (MFG, left) and anidulafungin (ANI, right). Shown are the parental strain CAF4-2 and 10 independent mutants lacking both copies of *CHT2*, *DUS4*, *RPS25B*, *UAP1* and orf19.970, as well as one copy of essential *URA7*, *RPO26*, *HAS1*, *CKS1*, and orf19.4149.1. Names, genotypes of strains and MIC_90_ are indicated on the right. The assay was conducted according to CLSI methods in RPMI 1640 medium with 2% glucose. The assay included a maximum caspofungin concentration of 2 μg/mL and 2-fold serial dilutions. A total of 10^3^ cells were inoculated into each well in four technical replicates and the tray was incubated at 35°C for 48 h. Control wells without the drug or without cells were included. The no-cell control was used to subtract the background. The no-drug control was used for normalization. The color bar for percent growth is presented below the heap map.

**FIG S3**. Broth microdilution assay of 12 Ch5 KOs against caspofungin (CAS). The heat map shows the similar growth of KOs vs their parental CAF4-2. Shown are the parental strain CAF4-2 and 12 independent mutants from Ch5 lacking both copies of orf19.4234, orf19.3220, orf19.3921, orf19.4150, orf19.3914, orf19.583, *TRY3* and *CKB2*, as well as one copy of essential orf19.969, orf19.3970, orf19.4149 and orf19.2639.1. Names, genotypes of strains and MIC_90_ are indicated on the right. The assay was conducted according to CLSI methods in RPMI 1640 medium with 2% glucose. The assay included a maximum CAS concentration of 2 μg/mL and 2-fold serial dilutions. A total of 10^3^ cells were inoculated into each well in four technical replicates and the tray was incubated at 35°C for 48 h. Control wells without the drug or without cells were included. The no-cell control was used to subtract the background. The no-drug control was used for normalization. The color bar for percent growth is presented below the heap map.

**FIG S4**. Susceptibility of 12 KOs from Ch5 to anidulafungin (ANI) and caspofungin (CAS) as determined with spot assay. Shown is growth of *CKB2*, *TRY3*, orf19.4234, orf19.3220, orf19.3914, orf19.583, orf19.4150 and orf19.3921 lacking both copies of gene and growth of essential orf19.969, orf19.2639.1, orf19.4149 and orf19.3970 lacking one copy of gene vs the parental strain CAF4-2. Control YPD and YPD supplemented with ANI or CAS are indicated. Also indicated is the time of incubation at 37º C. Strains are indicated on the left. Names of three KOs, which grow better than parental CAF4-2 are in frames.
