# Supplementary material for: Identification of 10 genes on Candida albicans chromosome 5 that control surface exposure of the immunogenic cell wall epitope β-glucan and cell wall remodeling in caspofungin-adapted mutants
Source: Microbiol Spectr. 2023 Nov 15;11(6):e03295-23. doi: 10.1128/spectrum.03295-23 (PMC10714753; doi:10.1128/spectrum.03295-23)
Supplement: Table S1 — List of primers and plasmids used in the study. [file spectrum.03295-23-s0006.pdf]

SUPPLEMENTAL MATERIALS

Table S1. List of primers and plasmids used in the study

**Primer purpose**	**Gene**	**Primer name and sequence**
Amplification of *CaCas9* cassette	*CaCas9*	CaCas9 F, ATCTCATTAGATTTGGAACTTGTGGGGTT<br>CaCas9 R, TTCGAGCGTCCCAAAACCTTCT
Amplification of SNR52 promoter and sgRNA scaffold		SNR52 F, AAGAAAGAAAGAAAACCAGGAGTGAA<br>sgRNA R, ACAAATATTTAAACTCGGGACCTGG
Amplification of final sgRNA expression cassette		SNR52/N F, GCGGCCGCAAGTGATTAGACT<br>sgRNA/N R, GCAGCTCAGTGATTAAGAGTAAAGAT GG
Amplification of sgRNA promoter and *SNR52* scaffold with overlapping ORF/gene guide sequence	*CHT2* (orf19.3895)	sgRNA/F_19.3895, GGTGTTAGAACCTGAACCAGGTTTTAGAGCTAGAAATAGCAAGTTAAA<br>SNR52/R_19.3895, CTGGTTCAGGTTCTAACACCCAAATTAAAAATAGTTTACGCAAGTC

Same as above	*URA7*<br>(orf19.3941)	SNR52/R_19.3941,<br>TCGGTGACATTGAAAGTGCTCAAATTAAAAAT<br>AGTTTACGCAAGTC<br>sgRNA/F_19.3941,<br>AGCACTTTCAATGTCACCGAGTTTTAGAGCTAG<br>AAATAGCAAGTTAAA
Same as above	*RPO26*<br>(orf19.2643)	SNR52/R_19.2643,<br>CCATTTGCTGATGGATCGTCCAAATTAAAAAT<br>AGTTTACGCAAGTC<br>sgRNA/F_19.2643,<br>GACGATCCATCAGCAAATGGGTTTTAGAGCTA<br>GAAATAGCAAGTTAAA
Same as above	*RPS25B*<br>(orf19.6663)	SNR52/R_19.6663,<br>CCGCTTTAGCTGGTGGTAAACAAATTAAAAAT<br>AGTTTACGCAAGTC<br>sgRNA/F_19. 6663,<br>TTTACCACCAGCTAAAGCGGGTTTTAGAGCTA<br>GAAATAGCAAGTTAAA
		SNR52/R_19.3962,<br>CAGGGAGATTATTGGATCATCAAATTAAAAAT<br>AGTTTACGCAAGTC

Same as above	*HAS1* (orf19.3962)	sgRNA/F_19. 3962, ATGATCCAATAATCTCCCTGGTTTTAGAGCTAGAAATAGCAAGTTAAA
Same as above	*DUS4* (orf19.966)	SNR52/R_19.966, CAGTTGATACCTATACCATCCAAATTAAAAATAGTTTACGCAAGTC<br><br>sgRNA/F_19. 966, GATGGTATAGGTATCAACTGGTTTTAGAGCTAGAAATAGCAAGTTAAA
Same as above	*CKS1* (orf19.1282)	SNR52/R_19.1282, CACGATTACTTTAATCAAGACAAATTAAAAATAGTTTACGCAAGTC<br><br>sgRNA/F_19.1282, TCTTGATTAAAGTAATCGTGGTTTTAGAGCTAGAAATAGCAAGTTAAA
Same as above	*UAP1* (orf19.4265)	SNR52/R_19.4265, TTGGGAGCACTTGAACCTAACAAATTAAAAATAGTTTACGCAAGTC

		sgRNA/F_19.4265,<br>TTAGGTTCAAGTGCTCCCAAGTTTTAGAGCTAG<br>AAATAGCAAGTTAAA
Same as above	orf19.4149.1	SNR52/R_19.4149.1,<br>TTCGTCATAGTATTTCATTCCAAATTAAAAATA<br>GTTTACGCAAGTC<br><br>sgRNA/F_19.4149.1,<br>GAATGAAATACTATGACGAAGTTTTAGAGCTA<br>GAAATAGCAAGTTAAA
Same as above	orf19.970	SNR52/R_19.970,<br>CCATGCATCCTTGGAACTTACAAATTAAAAAT<br>AGTTTACGCAAGTC<br><br>sgRNA/F_19.970,<br>TAAGTTCCAAGGATGCATGGGTTTTAGAGCTA<br>GAAATAGCAAGTTAAA
Same as above	*CKB2*<br>(orf19.4297)	SNR52/R_19.4297,<br><br>CGTCAAATTGAATCGGTCTCCAAATTAAAAAT<br>AGTTTACGCAAGTC<br><br>sgRNA/F_19.4297,

		GAGACCGATTCAATTTGACGGTTTTAGAGCTA GAAATAGCAAGTTAAA
Same as above	*TRY3* (orf19.1971)	SNR52/R_19.1971, TCGTATAATCTTCACTAGAACAAATTAAAAAT AGTTTACGCAAGTC sgRNA/F_19.1971, TTCTAGTGAAGATTATACGAGTTTTAGAGCTAG AAATAGCAAGTTAAA
Same as above	Orf19.4234	SNR52/R_19.4234, CTCCATTCACAGTAGTGTTGCAAATTAAAAAT AGTTTACGCAAGTC sgRNA/F_19. 4234, CAACACTACTGTGAATGGAGGTTTTAGAGCTA GAAATAGCAAGTTAAA
		SNR52/R_19.3220, CCCTTCTTTTTGTTAGTGTTCAAATTAAAAATA GTTTACGCAAGTC

Same as above	Orf19.3220	sgRNA/F_19. 3220,<br><br>AACACTAACAAAAAGAAGGGGTTTTAGAGCTA<br>GAAATAGCAAGTTAAA
Same as above	Orf19.969	SNR52/R_19.969,<br><br>CACATTAATGACCATATAATCAAATTAAAAAT<br>AGTTTACGCAAGTC<br><br>sgRNA/F_19. 969,<br><br>ATTATATGGTCATTAATGTGGTTTTAGAGCTAG<br>AAATAGCAAGTTAAA
Same as above	Orf19.3914	SNR52/R_19.3914,<br><br>TGTTCTTTCTTTTTATTTCTCAAATTAAAAATAG<br>TTTACGCAAGTC<br><br>sgRNA/F_19. 3914,<br><br>AGAAATAAAAAGAAAGAACAGTTTTAGAGCTA<br>GAAATAGCAAGTTAAA
		SNR52/R_19.583,<br><br>CGGTCATGTTTTTCCACCCACAAATTAAAAATA<br>GTTTACGCAAGTC

Same as above	Orf19.583	sgRNA/F_19. 583,<br><br>TGGGTGGAAAAACATGACCGGTTTTAGAGCTA<br>GAAATAGCAAGTTAAA
Same as above	Orf19.2639.1	SNR52/R_19.2639.1,<br><br>TCCTGTTTTGGTGCATTAGACAAATTAAAAATA<br>GTTTACGCAAGTC<br><br>sgRNA/F_19. 2639.1,<br><br>TCTAATGCACCAAAACAGGAGTTTTAGAGCTA<br>GAAATAGCAAGTTAAA
Same as above	Orf19.4150	SNR52/R_19.4150,<br><br>TGGTTTAATTTTTACTTTACCAAATTAAAAATA<br>GTTTACGCAAGTC<br><br>sgRNA/F_19.4150,<br><br>GTAAAGTAAAAATTAAACCAGTTTTAGAGCTA<br>GAAATAGCAAGTTAAA
		SNR52/R_19.3921,<br><br>CCATGCACGGTATGTCACATCAAATTAAAAAT<br>AGTTTACGCAAGTC

Same as above	Orf19.3921	sgRNA/F_19. 3921,<br><br>ATGTGACATACCGTGCATGGGTTTTAGAGCTA<br>GAAATAGCAAGTTAAA
Same as above	Orf19.4149	SNR52/R_19.4149,<br><br>CCTTAATACAGAAGTTAAAGCAAATTAAAAAT<br>AGTTTACGCAAGTC<br><br>sgRNA/F_19. 4149,<br><br>CTTTAACTTCTGTATTAAGGGTTTTAGAGCTAG<br>AAATAGCAAGTTAAA
Same as above	Orf19.3970	SNR52/R_19.3970,<br><br>CAATGCGAATAATCCCACTTCAAATTAAAAAT<br>AGTTTACGCAAGTC<br><br>sgRNA/F_19. 3970,<br><br>AAGTGGGATTATTCGCATTGGTTTTAGAGCTAG<br>AAATAGCAAGTTAAA
		Orf19.3895F,<br><br>TCTTCAAATCCAATTTAGTCTTTCGTTCTTATTT<br>AATTTTATACCCTTAACAAAACAAGCCAAAAA

Amplification of ORF/gene deletion cassette with NAT1	*CHT2* (orf19.3895)	GAAATAAAAGTATCGATGATTCATCCCATTCATTCCATC<br>Orf19.3895R, GTCTTAATAACTATTTGAGGGTTTTTTTATATATCAATACAAAAAGAAAAAATATTAGAGTAAACAAGAGGTTAATTCAGCCGCTCTAGAACTAGTGGATCT
Same as above	*URA7* (orf19.3941)	Orf19.3941F, TATTTTTTTGTATCAACGAATCAACAATAACAACAATAAGTACTTTTTAATTCCCTCCATCTCTCTATTAGCAAGTAATCGATGATTCATCCCATTCATTCCATC<br>Orf19.3941R, CATTACTACCTTATATACACATGACAATTAAGGAATAAAATCAATAAAAAAAAACAGCCAAAAGGAAATCACTTACTAATGCCGCTCTAGAACTAGTGGATCT
		Orf19.2643F, ATCTATCATCAACAAAAACCTCAAATCATCTAACTAATTCCGCCTCCCCCCCTTTATAATTTCAA

Same as above	*RPO26* (orf19.2643)	TACAAAACTTACATAGATGATTCATCCCATTCATTCCATC<br><br>Orf19.2643R,<br>TCTCTTGGGTTAACTTGGTCTCCTTTTTCTTATTCCTTTAACAATTCGTCTACTCCTGGGTTAATCAATAGTTCTATCGTGCCGCTCTAGAACTAGTGGATCT
Same as above	*RPS25B* (orf19.6663)	Orf19. 6663F,<br>GAATCAGTTATTCCCCTACGGCAGTGAATGAAAAATTCTTATTCAAGTCTAAGTATACTAACTAGTACAATTTTTTTAGAGATGATTCATCCCATTCATTCCATC<br><br>Orf19. 6663R,<br>TCATTTATTCTTTAGTAACATTAATAAAGAACAAGATAAATTATAAAATAACCATTAGATATAGATATTTTTTAATTAATGCCGCTCTAGAACTAGTGGATCT
		Orf19. 3962F,<br>ATTTTTTTTTCCAGGCTTTAGTTGATGAGATGACCTGATACATCTTACTCTTCACAACTGTTTCTT

Same as above	*HAS1* (orf19.3962)	ACCAACAACAAAAAGATGATTCATCCCATTCATTCCATC<br>Orf19. 3962R, GGAATATGGTACTGTTGATACAATGAATTATCACTATTATATTTCTATATAAAGATATGACCTATTAAAACTTATGAGTCGCCGCTCTAGAACTAGTGGATCT
Same as above	*DUS4* (orf19.966)	Orf19. 966F, TAAGATGAGCTTAAATATGGGAGAAAAAGAAATCTACATTTTTTTTTCATCACATCAGGTTAATACCAACTACCAAAAGATGATTCATCCCATTCATTCCATC<br>Orf19. 966R, TTAACAACAAGACTAGTAGTGTTTCACTAGCAAATATAGAAATGCATTAAATTAGTTATAGAAATTAAAAACAAAAGTACGCCGCTCTAGAACTAGTGGATCT
		Orf19. 1282F, CAATTAGTTCTTTTTTTTCATTTGTTTCCAGAGTTTAGGAAGACTACCATTTTACAATTTTCAATTC

Same as above	*CKS1* (orf19.1282)	AAATATTTTCCCAGATGATTCATCCCATTCATTCCATC<br>Orf19. 1282R, CTATTTCTTTCCTTTTTATGTTTTAATTCCAGTGTTAACTTTAAAACCGATTCTATAGTATTCAAATATAGTTAATCTTTGCCGCTCTAGAACTAGTGGATCT
Same as above	*UAP1* (orf19.4265)	Orf19. 4265F, CCAACAAAACTAGTTTTATTAATATTGCTGAAATAGAAAGTAAAAGAAATCAGCAACTTCTTACTATTATTATTATTATCGATGATTCATCCCATTCATTCCATC<br>Orf19. 4265R, TATATAATAAATAGCATAGCGCACATTATATTATATTATATTATATTATATTATGACTTTAGTAACTAAAAAAGTTTACAGCCGCTCTAGAACTAGTGGATCT
		Orf19.4149.1F, AACCAAAAAAAAAAAATAACTGAAATTTTTTTCAATAGTATTAGAGTATATTCTTAGACTGTATC

Same as above	orf19.4149.1	GAGCATTAAGACACCGATGATTCATCCCATTC ATTCCATC<br>Orf19.4149.1R, AAGAACCTTCAATGGCAGCTTTTGGGGTTTTGA AACCTAAACCAACATCTTTATACCATCTCTTGG TTTTGCCGCTCTAGAACTAGTGGATCTCTTGTT GGCT
Same as above	orf19.970	Orf19.970F, AATCTAACTCCTCCAATAATAGTTATACATAAT ATAATTTTACAAAACCTTCTTTTTTTTTTTAAAG TTTATTAACAACTGATGATTCATCCCATTCATT CCATC<br>Orf19.970R, GCAATATTGTCGAAGCATTTATTAGATCCTAAA GAAAATAAGCAAGATAATGAACCTACGGTAAA GAAACAGAAATTGAGGCCGCTCTAGAACTAGT GGATCT
		Orf19.4297F, GTTAATATTAAATATACCAAATAACTAATTCTT CTATTTTTTTTTTTGTTTTATTTCAGATTTACTAA

Same as above	*CKB2*<br>(orf19.4297)	GTTATAACAAAAGGATGATTCATCCCATTCATTCCATC<br>Orf19.4297R,<br>AT ACCAATATAGCAGCAAAAAAGTTTATTGAAGTACTCTAGTATTTACAAAACTGGTTGTGGTAGTGGTGGGACGTGATGGCCGCTCTAGAACTAGTGGATCT
Same as above	*TRY3*<br>(orf19.1971)	Orf19. 1971F,<br>TTCTAACAGACTATATTTCCCCCCCCCATCCCCTACCAAGATCAGTCGATTGGTTATGATTTGAGATATAGCACTCCATAGATGATTCATCCCATTCATTCCATC<br>Orf19. 1971R,<br>ATTTACACCGTAAAACTCTCATAAACACCACTCAAAACGAACATTTTATTAAACCAATTATAAACTTACTATTTATGTATGCCGCTCTAGAACTAGTGGATCT
		Orf19. 4234F,<br>TTCAGAGCGCATCGCGGAACAAAAAAAGAAATCTACAACTCAAAAGATACTTCCATAGAACTCA

Same as above	Orf19.4234	TCCCGACATACTACTAGATGATTCATCCCATTC ATTCCATC<br>Orf19. 4234R,<br>TTGAAATTTTAGAGTTTTTTTCCAATAACTATA GTGTAAATAAACGATTTTCTCTTTTTTTTTTTAT ATATATTGCTTTAGCCGCTCTAGAACTAGTGGA TCT
Same as above	Orf19.3220	Orf19. 3220F,<br>TTTCTTTTTCGGTGTAAAACCACATCTCATCTC ATTGCTTTAATAACAACTACAATACACATAAC ACATAACACATAAAAGATGATTCATCCCATTC ATTCCATC<br>Orf19. 3220R,<br>ATCGTTTTTTTCTAAACTTTCCCCTTAAACCCTA TTTTTATATCCACCATTAACCCCCCCCCCTCTC CCTTTCTTCACTTGCCGCTCTAGAACTAGTGGA TCT
		Orf19. 969F,<br>CACCATTTAAACTCTTCTTATTCTTTTTGTAGTC GATTTTCTCTCTCAATCCCACAGTGTCCACGTT

Same as above	Orf19.969	GTCTTCCCTAAACGATGATTCATCCCATTCATTCCATC<br>Orf19. 969R,<br>AATAACATTTCATACATTTACAAACAATTTTTTAAGCTTCTCTTTACTCACAACATACTTAATACAAAAACACTCTTCTCGCCGCTCTAGAACTAGTGGATCT
Same as above	Orf19.3914	Orf19. 3914F,<br>ATAGCTTATTTGATCAAGAAATAAGAGTGGGTTCTTTCTTTTTTATACATAAATAATAGTCCATTTTAACTAATTTCATCGATGATTCATCCCATTCATTCCATC<br>Orf19. 3914R,<br>GTTTCAAAAATGTGTGAAAACACTTTATATGTACACTTATATAGCTACATACCCTGAAAACTTCTGGTGTATCAGCACATGCCGCTCTAGAACTAGTGGATCT
		Orf19. 583F,<br>TTAGGGATAAAACGGGAAACGTTATAAAAATTTCTTTTTTGTTGCACCACTTTCTTTTTTATATTT

Same as above	Orf19.583	TTTTTTTTTCACCAGATGATTCATCCCATTCATT<br>CCATC<br><br>Orf19. 583R,<br><br>CTTTTTAATGGGCATAAAACTTCATTTATTCTG<br>CTTTTTTTTTTGGCTTATTATCCGTGTACACAAA<br>CATACAGTACTGCGCCGCTCTAGAACTAGTGG<br>ATCT
Same as above	Orf19.2639.1	Orf19. 2639.1F,<br><br>TGTTTTGAGGAAGAACGAGAAAAAAAAACTCT<br>TGGAGTAATTATTTCATTGACAGTGAATCAATA<br>AACAACCAATCAACCGATGATTCATCCCATTC<br>ATTCCATC<br><br>Orf19. 2639.1R,<br><br>TATGACGTGTATATATATATATTATATTACTAA<br>TACAAAAAAACAACATATATTTATGCCTTGAG<br>TTTATTCTCCCCTCAGCCGCTCTAGAACTAGTG<br>GATCT
		Orf19.4150F,<br><br>ATACATATGCTTTAATTTAACCTTCCCGCCTTT<br>CTTTTCTTCTTTTTGAATTATATCGATTTTAAAA

Same as above	Orf19.4150	ACTACACTTCATCGATGATTCATCCCATTCATTCCATC<br>Orf19.4150R,<br>TACTATATGTTTACTTACCAGTTGTATTGAGTACATATTTATATAATGGAGGAACCAATGTAATAAATATTAGGAAATGTGCCGCTCTAGAACTAGTGGATCT
Same as above	Orf19.3921	Orf19. 3921F,<br>TTTTTTTTTTGCGCTTATCGAAGTGATTTTTTTTTTCTTTCATTTTCTTCCTTTCTTCTTCTTTTCCAGATTCAACTCCAGATGATTCATCCCATTCATTCCATC<br>Orf19. 3921R,<br>AATATAGAACAGGAAAAAAGAAAATAGAACATGACGAATTATAGTCTATATAGAATCTAATAAATGCGTTTCCCCATTTCGCCGCTCTAGAACTAGTGGATCT
		Orf19. 4149F,<br>ATTATATAAATATGTACTCAATACAACTGGTAAGTAAACATATAGTAAACACGTAGACATGGTA

Same as above	Orf19.4149	ATAAATACTTAATCACGATGATTCATCCCATTC ATTCCATC<br>Orf19. 4149R,<br>TCT GCT GGT GCT TCT AGA TCC AAG AAA TTC TCT AAA TTC TAA ACA AAA CCC CAA GAA GAT AAA AGA GAA TAA TCT AAT CCGCCGCTCTAGAACTAGTGGATCT
Same as above	Orf19.3970	Orf19. 3970F,<br>ATTTTCATTTTCTCAACAAAGATTACGATGAGA CAAAAAAAAAAAAAAATATACCAAACTCAT ATCAAGAGATGAATAAGATGATTCATCCCATT CATTCCATC<br>Orf19. 3970R,<br>TTTAACAAAAAAGTTGCAAACCTTATTATTATT ATTATCAATACTATTACTACTACCAAGATGGAC TTTATATTAAGGTTGCCGCTCTAGAACTAGTGG ATCT
	CHT2	Orf19.3895-fwd,<br>TTGATCCAAGATATCTCCAAACTTG<br>Orf19.3895-rev, GGTAACAGCAGAGGAAGTAGT

Confirmation of deletion of ORF/gene	(orf19.3895)	Flk19.3895-rev, TGAAGAACAATAACAAACCCATGTA
Same as above	*URA7* (orf19.3941)	Orf19.3941-fwd, ATCCTACTTTAATAATACCGTTTGTTTATTCAC<br>Orf19.3941-rev, ATGGATTAATGCAAAATTTTCGTTACC<br>Flk19.3941-rev, GAGGAATGTAAAATTGAAATTACTCTATAATC C
Same as above	*RPO26* (orf19.2643)	Orf19.2643-fwd, TGATTGCAAACTGGTAGTCG<br>Orf19.2643-rev, CTAGTTCCCAATATTCTTGCTCTTTC<br>Flk19.2643-rev, CATTGATTAAATACAATACAAAAGGGG
Same as above	*RPS25B* (orf19.6663)	Orf19. 6663-fwd, CAACAACAGTAATAGAAGTGACCAG<br>Orf19. 6663-rev, TCTGGCTAAAGAACCACCAAT<br>Flk19. 6663-rev, AAGGGAGATGTTGAATCAGGAA

Same as above	*HAS1* (orf19.3962)	Orf19. 3962-fwd, TCACCTCCCATATTACAGATGTTG<br>Orf19. 3962-rev, CAATAATTCAATTGCAGGAATTAAGAATGC<br>Flk19. 3962-rev, AGCAAAGATGATTTACGTGAACG
Same as above	*DUS4* (orf19.966)	Orf19. 966-fwd, GAGAAATTAACATAAGTGGGAGCTAC<br>Orf19. 966-rev, AACACCGTCTACACCTGTGTA<br>Flk19. 966-rev, ATTGTGGCCAATTACATCAAGG
Same as above	*CKS1* (orf19.1282)	Orf19. 1282-fwd, GATAGATATCAATTACTAATTTACCCTTGTTTTTTAC<br>Orf19. 1282-rev, CTGGAGCATGAGTTTCGTAATG<br>Flk19. 1282-rev, CAATGAGATTGGCTTATAAATAGTGTATAC
		Orf19.4265-fwd, CTTCCAACTAAACAACAACAAACAAC

Same as above	*UAP1* (orf19.4265)	Orf19.4265-rev, TTTGATGCCCTTAGAATTCAAATCATC<br>Flk19.4265-rev, GTTACTGTTGAAAAGATATATGAAAAATTTAGAG
Same as above	orf19.4149.1	Orf19.4149.1-fwd, GGTACACACAAACCAAGTGA<br>Orf19.4149.1-rev, TTTAGGATGAAGAATATCTTTTTAAACTCC<br>Flk19.4149.1-rev, TCTACATTGACCAACGGTGA
Same as above	orf19.970	Orf19.970-fwd, ATTGAGAGAGAAAATCGACTACAA<br>Orf19.970-rev, ATTTAGCACTTCCTCAGTAAGTTC<br>Flk19.970-rev, ATTTGCAGAATGGTATGTCGGA
Same as above	*CKB2* (orf19.4297)	Orf19.4297-fwd, ACTAAGAAGCCATTTCCATCACTAC<br>Orf19.4297-rev, GGAAATTCTGGAAAAACATTGCTGG<br>Flk19.4297-rev, GTGTTCTACAAGGTGCACCC

Same as above	*TRY3*<br>(orf19.1971)	Orf19. 1971-fwd,<br>CAACAACAACAACATTACTAAACCCGA<br>Orf19. 1971-rev,<br>TTT GAA ATA AAC TTC AGA GTC GAG ATA CAC<br>Flk19. 1971-rev,<br>TAGTAGCTACATAGAACATTTCAAGATTTCAAC
Same as above	Orf19.4234	Orf19. 4234-fwd,<br>TCGAGAGTGGAACTTATCTCG<br>Orf19. 4234-rev,<br>GT TTT GGT TTG CAC AAT CAC TGA<br>Flk19. 4234-rev,<br>CTCATTTGACAAAGATGTCTTGCC
		Orf19. 3220-fwd,<br>AAGACCTTAGCCGACACC<br>Orf19. 3220-rev,<br>ACC AAC ACT CAT TAT AGG AAT CCC

Same as above	Orf19.3220	Flk19. 3220-rev,<br>CTACTCATTTTTCATAACAAATAAACCTTG
Same as above	Orf19.969	Orf19. 969-fwd,<br>GTATAACTATTATTGGAGGAGTTAGATTTCATC<br>Orf19. 969-rev,<br>T GGT AGT CTC ATC TTC GTC ATC<br>Flk19. 969-rev,<br>GGGAAAGTCACTCACGAGA
Same as above	Orf19.3914	Orf19. 3914-fwd,<br>TCTATCTGCATTATCCGTTTGTG<br>Orf19. 3914-rev,<br>ATC AGG AAT TAA AGA TCC CGT TAA<br>Flk19. 3914-rev,<br>CAAGTATTACCGTGTGCATTAGG
		Orf19. 583-fwd,<br>CTAAACACCGACATGGAGTTGA<br>Orf19. 583-rev,<br>GTC CAA GAA CTG AAT TAA TGA ACT TTG

Same as above	Orf19.583	Flk19. 583-rev,<br><br>GTTACCTTCATGAACCAACTACTATAAA
Same as above	Orf19.2639.1	Orf19. 2639.1-fwd,<br><br>CCCAAATAAAGCTCTTTAACAATTGTG<br><br>Orf19. 2639.1-rev,<br><br>C CAC CGG TGA TAT AGT CAA TAA ACT<br><br>Flk19. 2639.1-rev,<br><br>GTATGGACAAAATCAGGTTGCG
Same as above	Orf19.4150	Orf19.4150-fwd,<br><br>AAGACAACAGCTAAAATTTTTTGGTCAGAACA<br>C<br><br>Orf19.4150-rev,<br><br>TGT CAA ATA ACT TTG TAA TTC AGC TCC<br>ATA TTC<br><br>Flk19.4150-rev,<br><br>GTAATATGAGAAAGTGTTTCTCTCTC CAGGG
		Orf19. 3921-fwd,<br><br>GTTGCACGTA GAATTATAGGTTTC

Same as above	Orf19.3921	Orf19. 3921-rev,<br>GAC TCC ACC AAA TGC AGC<br>Flk19. 3921-rev,<br>AACAAACCCAGCAACAACC
Same as above	Orf19.4149	Orf19. 4149-fwd,<br>ATTTGATTAA ATTGTTAGTT GAATGGGGGT<br>Orf19. 4149-rev,<br>ACA ACA ACA AGA AAA ACA AAA CAT ATG CCT<br>Flk19. 4149-rev,<br>CC ATC ATT ATC AGA AGA GAT TAC TTG CAT
Same as above	Orf19.3970	Orf19. 3970-fwd,<br>TGATCCACAATGAAGTCAGTTG<br>Orf19. 3970-rev,<br>ATT GGA TGT TAA ATT CGA AGC AGT<br>Flk19. 3970-rev,<br>CTATTCTTGTAACTCG TGGGC

Confirmation of deletion of ORF/gene	NAT1	NAT1 RP4, CACAGACGCGTTGAATTGT
**Real-Time qRT-PCR primers**		
Amplification of *CaACT1* gene	*ACT1*	ACT1 FWD Set 1, ACTACCATGTTCCCAGGTATTG<br><br>ACT1 REV Set 1, CCACCAATCCAGACAGAGTATT
Amplification of *CaFKS1* gene	*FKS1*	FKS1 FWD Set 1, GGATATCAAGACCAAGCCAACTA<br><br>FKS1 REV Set 1, CCAGGAGTTTGACCACCATAA
Amplification of *CaFKS2* gene	*FKS2*	FKS2 FWD Set 1, CTAGCAGTCGCCAATCATGTA<br><br>FKS2 REV Set 1, CCGATAATGCAAACCCAAGAAC
Amplification of *CaFKS3* gene	*FKS3*	FKS3 FWD Set 1, AGCTTGGTGCCCTGAAA<br><br>FKS3 REV Set 1, GTTGCTGACATTATCGTCTTGG
**Plasmids**	**Description and Purpose**	
pV1093	CaCas9/gRNA cassette carrying ampicillin resistance gene, amplification of CaCas9/gRNA cassette	
pJK863	CaNAT1-FLP cassette carrying nourseothricin resistance gene, amplification of NAT cassette	
